# A Boundary-Local Mass Cocycle and the Mass of Asymptotically Hyperbolic Manifolds

**DOI:** 10.1007/s00220-024-05079-3

**Published:** 2024-09-16

**Authors:** Andreas Čap, A. Rod Gover

**Affiliations:** 1 Faculty of Mathematics, University of Vienna, Oskar–Morgenstern–Platz 1, 1090 Vienna, Austria; 2 Department of Mathematics, The University of Auckland, Private Bag 92019, Auckland, 1142 New Zealand

**Keywords:** primary: 53A55, secondary: 53C18, 53C25, 53C80, 83C30, 83C60

## Abstract

We construct a cocycle that, for a given *n*-manifold, maps a pair of asymptotically locally hyperbolic (ALH) metrics to a tractor-valued $(n-1)$-form field on the conformal infinity. This requires the metrics to be asymptotically related to a given order that depends on the dimension. It then provides a local geometric quantity on the boundary that is naturally associated to the pair and can be interpreted as a relative energy-momentum density. It is distinguished as a geometric object by its property of being invariant under suitable diffeomorphisms fixing the boundary, and that act on (either) one of the argument metrics. Specialising to the case of an ALH metric *h* that is suitably asymptotically related to a locally hyperbolic conformally compact metric, we show that the cocycle determines an absolute invariant *c*(*h*), which still is local in nature. This tractor-valued $(n-1)$-form field on the conformal infinity is canonically associated to *h* (i.e. is not dependent on other choices) and is equivariant under the appropriate diffeomorphisms. Finally specialising further to the case that the boundary is a sphere and that a metric *h* is asymptotically related to a hyperbolic metric on the interior, we show that the invariant *c*(*h*) can be integrated over the boundary. The result pairs with solutions of the KID (Killing initial data) equation to recover the known description of hyperbolic mass integrals of Wang, and Chruściel–Herzlich.

## Introduction

In general relativity, and a number of related mathematical studies, the notion of a “mass” invariant for relevant geometric manifolds is extremely important and heavily studied [2, 18, 20]. In general defining and interpreting a suitable notion of mass is not straightforward. For so-called asymptotically flat manifolds, the Arnowitt–Deser–Misner (ADM) energy-momentum is well established and is usually accepted as the correct definition. Motivated by the desire to define and study mass in other settings Wang [22] and Chruściel and Herzlich [9] introduced a notion of mass integrals and “energy-momentum” for Riemannian manifolds that, in a suitable sense, are asymptotically hyperbolic. These have immediate applications for a class of static spacetimes. The mass integrals are parametrized by solutions of the KID (Killing initial data) equation for the hyperbolic background. Denoting by *n* the interior dimension, these solutions form an $n+1$-dimensional vector space that is endowed with a natural Lorentzian metric. This leads to the interpretation that the mass in the asymptotically hyperbolic setting is a vector in that $n+1$-dimensional space rather than just a number.

The aim of this article is to construct new invariants that capture a notion of mass density, in the setting of asymptotically hyperbolic metrics. These invariants are locally defined (as quantities on the boundary at infinity) as volume forms with values in the standard tractor bundle associated to a conformal structure on the boundary, which has rank $n+1$ and carries a natural Lorentzian bundle metric. In the special case of hyperbolic space as a background, we prove that these local quantities can be integrated to global parallel sections of the tractor bundle and then naturally recover the mass as introduced by Wang and Chruściel–Herzlich. The setting we work in is rather restrictive in some aspects but very general in other aspects. The main restriction is that we are working in a conformally compact setting, so we need strong assumptions on the order of asymptotics. On the other hand, underlying this is an arbitrary manifold with boundary with no restrictions on the topology of the boundary. We also allow a fairly general “background metric”; the core of our results only require a background metric that is asymptotically locally hyperbolic (ALH), as in Definition 2.2, part (1). The main invariant we construct is associated to a pair of ALH metrics (that are asymptotic to sufficient order), so it should be thought of as a *relative local mass* or as a *local mass difference*.

Our basic setting looks as follows. We start with an arbitrary manifold ${\overline{M}}$ of dimension $n\ge 3$, with boundary $\partial M$ and interior *M*. Given two conformally compact metrics *g* and *h* on *M*, there is a well defined notion of *g* and *h* approaching each other asymptotically to certain orders towards the boundary. The actual order we need depends on the dimension and specializes to the order required in [22] on hyperbolic space. This defines an equivalence relation on conformally compact metrics and we consider one equivalence class ${\mathcal {G}}$ of such metrics. The only additional requirement at this point is that ${\mathcal {G}}$ consists of ALH-metrics. Since ${\mathcal {G}}$ consists of conformally compact metrics, each $g\in {\mathcal {G}}$ gives rise to a conformal structure on $\partial M$, called the conformal infinity of *g*. Moreover, the asymptotic condition used to define ${\mathcal {G}}$ is strong enough to ensure that all metrics in ${\mathcal {G}}$ lead to the same conformal infinity. Thus ${\mathcal {G}}$ canonically determines a conformal structure $[{\mathcal {G}}_\infty ]$ on $\partial M$.

This last fact is crucial for the further development, since the invariants we construct are geometric objects for this conformal structure on $\partial M$ (and a slightly stronger structure in case that $\dim ({\overline{M}})=3$). Indeed, the conformal structure $[\mathcal G_\infty ]$ canonically determines the so-called *standard tractor bundle* ${\mathcal {T}}\partial M\rightarrow \partial M$. This is a vector bundle of rank $n+1$ endowed with a Lorentzian bundle metric and a metric linear connection [1]. We construct *cocycles* *c* that associate to each pair of metrics $g,h\in {\mathcal {G}}$ a tractor-valued $(n-1)$-form $c(g,h)\in \Omega ^{n-1}(\partial M,{\mathcal {T}}\partial M)$, with the property that $c(h,g)=-c(g,h)$ and $c(g,k)=c(g,h)+c(h,k)$ for all $g,h,k\in {\mathcal {G}}$. These cocycles are the basic “relative local masses” we consider.

The idea of the construction is that any metric $g\in {\mathcal {G}}$ determines a conformal class on ${\overline{M}}$ (since the conformal class of *g* on *M* extends to the boundary by conformal compactness) and this restricts to $[{\mathcal {G}}_\infty ]$ at the boundary. Working with this conformal structure, we can use standard techniques of tractor calculus, and an instance of what is called a BGG splitting operator, to associate to any $h\in {\mathcal {G}}$ a one-form with values in the tractor bundle ${\mathcal {T}}{\overline{M}}$. The Hodge dual (with respect to *g*) of this one-form is then shown to admit a smooth extension of the boundary whose boundary value is defined to be *c*(*g*, *h*), see Propositions 3.1 and 3.5. The proof that this actually defines a cocycle needs some care because of the use of different conformal structures on ${\overline{M}}$, but is otherwise straightforward. The idea to seek objects valued in ${\mathcal {T}}{\overline{M}}$ was motivated by a desire to link to the KID (Killing initial data) equation, and its solutions, which (as we discuss later), has a nice interpretation in the tractor picture. This, with the conformal compactification, leads to the need to use conformal tractors and tools that are conformally invariant so that they extend in a simple and practical way to the boundary. The BGG splitting operators we use are then the unique conformally invariant operators available to extract the required jet data and include this in appropriate bundles. The use of tractors and conformally invariant operators is also crucial for allowing us to work locally (along the boundary), which is an essential feature of our approach.

Initially, this leads to a two parameter family of cocycles since there are two constructions of the above type, one depending on the trace of the difference $g-h$, the other on its trace-free part. Our constructions do not require charts or coordinates, so, in that sense, are automatically geometric in nature. The constructions also readily imply equivariancy with respect to the appropriate diffeomorphisms. If $\Phi$ is a diffeomorphism of ${\overline{M}}$ which preserves the class ${\mathcal {G}}$ (in an obvious sense) then by definition the restriction $\Phi |_{\partial M}$ is a conformal isometry for $[{\mathcal {G}}_\infty ]$. Therefore, it naturally acts on sections of ${\mathcal {T}}\partial M$ (and of course on forms on $\partial M$) and for any of the cocycles $c(\Phi ^*g,\Phi ^*h)=(\Phi |_{\partial M})^*c(g,h)$, see Proposition 3.7.

There is a much more subtle compatibility condition with diffeomorphisms, however. Indeed, consider a diffeomorphism $\Psi$ that is compatible with $[{\mathcal {G}}_\infty ]$ and suppose that $\Psi |_{\partial M}={\text {id}}_{\partial M}$. Then it turns out that $\Psi$ is asymptotic to the identity of order $n+1$ in a well-defined sense, see Sect. 3.4 and Theorem 3.11. The main technical result of our article then is that there is a unique ratio of the two parameters for our cocycles, which ensures that $c(g,\Psi ^*h)=c(g,h)$ for any $\Psi$ which is asymptotic to the identity of order $n+1$. Hence, up to an overall normalization we obtain a unique cocycle *c* which has this invariance property in addition to the equivariancy property mentioned above. This proof is based on an idea in [10] that shows that the action of diffeomorphisms that are asymptotic to the identity can be absorbed into a geometric condition relating the two metrics (and an adapted defining function for one of them). The key feature of this property is that it holds in the general setting of a class of ALH metrics on a manifold with boundary. In the special cases for which we obtain invariants of single metrics, this property enables us to prove equivariancy of such invariants under diffeomorphisms preserving ${\mathcal {G}}$, see below.

To pass from our cocycles to invariants of a single metric, one has to go to specific situations in which ${\mathcal {G}}$ contains particularly nice metrics. We only discuss the case that $\mathcal G$ locally contains metrics that are hyperbolic, i.e. have constant sectional curvature $-1$. This of course implies that $(\partial M,[{\mathcal {G}}_\infty ])$ is conformally flat, but it does not impose further restrictions on the topology of $\partial M$, see Sect. 3.8. Under this assumption, we show that, for a cocycle from our one-parameter family and a metric $h\in {\mathcal {G}}$, all local hyperbolic metrics $g\in {\mathcal {G}}$ locally lead to the same tractor-valued form *c*(*g*, *h*). These local forms then piece together to define an object $c(h)\in \Omega ^{n-1}(\partial M,\mathcal T\partial M)$ that is canonically associated to *h*. We prove that this is equivariant under diffeomorphisms preserving the class ${\mathcal {G}}$, see Theorem 3.17.

As a last step, we prove that, in the conformally compact setting, the AH mass as introduced in [9] can be obtained by integrating our invariant. Thus we have to specialize to the case that ${\overline{M}}$ is an open neighborhood of the boundary sphere in the closed unit ball and ${\mathcal {G}}$ contains the restriction of the Poincaré metric. This implies that $\partial M=S^{n-1}$ and $[{\mathcal {G}}_\infty ]$ is the round conformal structure and thus the standard tractor bundle $\mathcal TS^{n-1}$ can be globally trivialized by parallel sections. Hence $\mathcal TS^{n-1}$—valued $(n-1)$-forms can be integrated to global parallel sections of $\mathcal TS^{n-1}$ (say by expanding in any globally parallel frame and then integrating the coefficient forms). Now it is well known how to make the trivialization of $\mathcal TS^{n-1}$ explicit, and we show that there is a particularly nice way to do this using the conformal class [*g*] on ${\overline{M}}$ determined by the Poincaré metric. Via boundary values of parallel tractors in the interior, the parallel sections of $\mathcal TS^{n-1}$ turn out to be parametrized by the solutions of the KID (Killing initial data) equation (3.25) on *M*. But these solutions exactly parameterize the mass integrals used to define the AH mass in the style of [9], see [19]. This last fact was our original motivation to look for a tractor interpretation of the AH mass.

Now a solution *V* of the KID equation determines a parallel section $s_V$ of $\mathcal TS^{n-1}$ and we can proceed as follows. Given $h\in {\mathcal {G}}$, we can form the invariant $c(h)\in \Omega ^{n-1}(S^{n-1},\mathcal TS^{n-1})$ and integrate it to a parallel section $\int _{S^{n-1}}c(h)$ of $\mathcal TS^{n-1}$. This can then be paired, via the tractor metric, with $s_V$. Analyzing the boundary behavior of the mass integral determined by *V*, we show in Theorem 3.18 that, after appropriate normalization, this pairing exactly recovers the mass integral, of [9], determined by *V*.

Throughout all manifolds, tensor fields, and related objects, will be taken to be smooth in the sense of $C^\infty$. For most results lower regularity would be sufficient, but we do not take that up here.

## Setup and Tractors

### Conformally compact metrics

Let ${\overline{M}}$ be a smooth manifold with boundary $\partial M$ and interior *M*. Recall that a *local defining function* for $\partial M$ is a smooth function $\rho :U\rightarrow {\mathbb {R}}_{\ge 0}$ defined on some open subset $U\subset {\overline{M}}$ such that $U\cap \partial M=\rho ^{-1}(\{0\})$ and such that $d\rho |_{U\cap \partial M}$ is nowhere vanishing. For two local defining functions $\rho$ and ${{\hat{\rho }}}$ defined on the same open set *U*, there is a smooth function $f:U\rightarrow {\mathbb {R}}_{>0}$ such that ${{\hat{\rho }}}=f\rho$. It is often convenient to write such positive function as $f=e^{{{\tilde{f}}}}$ for some smooth function ${{\tilde{f}}}$. This notion of defining functions extends, without problem, from functions to smooth sections of line bundles. One just has to replace $d\rho$ by the covariant derivative with respect to any linear connection, which is independent of the connection along the zero set of the section. In particular, taking the line bundle concerned to be a density bundle (as discussed above Proposition 2.1 below) leads to *defining densities* [6].

A pseudo-Riemannian metric *g* on *M* is called *conformally compact* if, for any point $x\in \partial M$, there is a local defining function $\rho$ for $\partial M$ defined on some neighborhood *U* of *x* such that $\rho ^2g$ admits a (sufficiently) smooth extension from $U\cap M$ to all of *U*, with non-degenerate boundary values. It is easy to see that this property is independent of the choice of defining function, so if *g* is conformally compact, then for any local defining function ${{\hat{\rho }}}$ for $\partial M$, the metric ${\hat{\rho }^2} g$ admits a smooth extension to the boundary. While the metric on the boundary, induced by such an extension, depends on the chosen defining function, such extensions are always conformally related. Hence a conformally compact metric on *M* gives rise to a well-defined conformal class on $\partial M$, which is called the *conformal infinity* of *g*. The model example of a conformally compact metric is the Poincaré ball model of hyperbolic space. Here ${\overline{M}}$ is the closed unit ball in ${\mathbb {R}}^{n}$ and one defines a metric *g* on the open unit ball as $\tfrac{4}{(1-|x|^2)^2}$ times the Euclidean metric, see [17]. The resulting conformal infinity is the conformal class of the round metric on $S^{n-1}$.

There is a conceptual description of conformally compact metrics in the language of conformal geometry: Since $\rho ^2g$ is conformal to *g* away from the boundary, it provides a (sufficiently) smooth extension of the conformal structure on *M* defined by *g* to all of ${\overline{M}}$. Conformally compact metrics can be neatly characterized in this picture via their volume densities. Recall that the volume density of *g* is nowhere vanishing, so one can form powers with arbitrary real exponents to obtain nowhere vanishing densities of all (non-zero) weights. Each of these densities is parallel for the connection on the appropriate density bundle induced by the Levi-Civita connection, and up to constant multiples, this is the only parallel section.

In the usual conventions of conformal geometry, see [1], the square of the top exterior power of the tangent bundle, that is $\otimes ^2(\Lambda ^nTM)$, is identified with a line bundle of *densities* of weight 2*n* that we denote ${\mathcal {E}}[2n]$. This is oriented and hence trivial, so there is a standard notion of its roots. With these conventions, on an *n*-manifold a metric *g* determines a volume density ${\text {vol}}_g$ that has conformal weight $-n$, meaning ${\text {vol}}_g\in \Gamma ({\mathcal {E}}[-n])$. Rescaling *g* to $\rho ^2g$, we get ${\text {vol}}_{\rho ^2g}=\rho ^n{\text {vol}}_g$ and thus ${\text {vol}}_{g}^{-1/n}=\rho {\text {vol}}_{\rho ^2g}^{-1/n}\in \Gamma ({\mathcal {E}}[1])$. Assuming that *g* is conformally compact, ${\text {vol}}_{\rho ^2g}^{-1/n}$ is smooth up to the boundary and nowhere vanishing, so this equation shows that ${\text {vol}}_{g}^{-1/n}\in \Gamma ({\mathcal {E}}[1])$ is a defining density for $\partial M$. Similar arguments prove the converse, which leads to the following result.

#### Proposition 2.1

Let ${\overline{M}}$ be a smooth manifold with boundary $\partial M$ and interior *M*, which is endowed with a conformal structure *c*. Then a metric *g* on *M* which lies in $c|_M$ is conformally compact if an only if any non-zero section $\sigma \in \Gamma ({\mathcal {E}}[1]|_M)$, which is parallel for the Levi-Civita connection of *g* extends by 0 to a defining density for $\partial M$.

### ALH-metrics and adapted defining functions

Consider a conformally compact metric *g* and for a local defining function $\rho$ put ${\overline{g}}:=\rho ^2g$, and note that this is smooth and non-degenerate up to the boundary. Hence also the inverse metric ${\overline{g}}^{-1}$ is smooth up to the boundary, so ${\overline{g}}^{-1}(d\rho ,d\rho )$ a smooth function on the domain of definition of $\rho$. Moreover, since $d\rho$ is nowhere vanishing along the boundary ${\overline{g}}^{-1}(d\rho ,d\rho )$ has the same property. Replacing $\rho$ by ${{\hat{\rho }}}:=e^f\rho$, we obtain $d{{\hat{\rho }}}={{\hat{\rho }}} df+e^fd\rho$, which easily implies that the restriction of ${\overline{g}}^{-1}(d\rho ,d\rho )$ to $\partial M$ is independent of the defining function $\rho$.

So ${\overline{g}}^{-1}(d\rho ,d\rho )$ is an invariant of the metric *g*, and this is related to the asymptotic behavior of the curvature of *g*, see e.g. [17]. In particular, if ${\overline{g}}^{-1}(d\rho ,d\rho )|_{\partial M}\equiv 1$, then the sectional curvature of *g* is asymptotically constant $-1$. This justifies the terminology in the first part of the following definition and leads to a subclass of defining functions:

#### Definition 2.2

Consider a smooth manifold ${\overline{M}}=M\cup \partial M$ with boundary and a conformally compact metric *g* on *M*.

1. The metric *g* is called *asymptotically locally hyperbolic* (or an ALH-metric) if $(\rho ^2g)^{-1}(d\rho ,d\rho )|_{\partial M}$ is identically one.
2. Assume that *g* is an ALH-metric on *M* and that $U\subset {\overline{M}}$ (with $U\cap \partial M\ne \emptyset$ to be of interest). Then a local defining function $\rho$ for $\partial M$ defined on *U* is called *adapted to* *g* if the function $(\rho ^2\,g)^{-1}(d\rho ,d\rho )$ is identically one on some open neighborhood of $U\cap \partial M$.

#### Remark 2.3

Note that in the literature the terminology “asymptotically locally hyperbolic” is sometimes used for (various) more restrictive classes of geometry. Also the condition we have (1) is sometimes referred to as simply “asymptotically hyperbolic”. However the latter is also used for rather special settings where in particular the boundary is necessarily a sphere, hence our use here.

The existence of adapted defining functions, as in part (2) of Definition 2.2, can be established by solving an appropriate non-characteristic first order PDE. The following precise description of adapted defining functions is given in Lemma 2.1 of [17].

#### Proposition 2.4

Consider ${\overline{M}}=M\cup \partial M$ and an ALH metric *g* on *M*. Then for any choice of a representative metric *h* in the conformal infinity of *g*, there exists an adapted defining function $\rho$ for *g*, defined on an open neighborhood of $\partial M$ in ${\overline{M}}$, such that $\rho ^2g$ induces the metric *h* on $\partial M$. Moreover, for fixed *h*, the germ of $\rho$ along $\partial M$ is uniquely determined.

### The basic setup

Defining functions can be used to measure the asymptotic growth (or fall-off) of functions and more general geometric objects on the interior of a manifold with boundary. A fundamental property of defining functions is that for a function *f* that is smooth up to the boundary $f|_{\partial M}\equiv 0$ if and only if for any local defining function $\rho$ for $\partial M$, we obtain, on the domain of definition of $\rho$, $f=\rho f_1$ for a function $f_1$ that is smooth up to the boundary. We say that *f* is ${\mathcal {O}}(\rho )$ in this case, observing that this notion is actually independent of the specific defining function $\rho$. Similarly if, in such an expansion, $f_1$ also vanishes along the boundary then this fact does not depend on the choice of $\rho$, and in that case we say that *f* is ${\mathcal {O}}(\rho ^2)$. Inductively, one obtains the notion that *f* is ${\mathcal {O}}(\rho ^N)$ for any integer $N>0$, which again does not depend on the specific choice of defining function.

Given a smooth function $f:M\rightarrow {\mathbb {R}}$ and an integer $N>0$, we then say that *f* is ${{\mathcal {O}}} (\rho ^{-N})$ if locally around each $x\in \partial M$ we find a defining function $\rho$ such that $\rho ^Nf$ admits a smooth extension to the boundary. Again, the fact that such an extension exists is independent of the choice of defining function, as is vanishing of the boundary value of $\rho ^N f$ in some point. In points where the boundary value of $\rho ^Nf$ is nonzero, the actual value does depend on the choice of $\rho$, however.

For two functions $f_1,f_2:{\overline{M}}\rightarrow {\mathbb {R}}$ and $N>0$, we write $f_1\sim _N f_2$ if $f_1-f_2$ is ${\mathcal {O}}(\rho ^N)$. By definition, this means that, on the domain of a local defining function $\rho$, we can write $f_2=f_1+\rho ^Nf$ for some function *f* that is smooth up to the boundary. Observe that this defines an equivalence relation.

All this extends without problems to tensor fields of arbitrary (fixed) type. This can be seen immediately from looking at coordinate functions in (boundary) charts. So for $N>0$, a tensor field *t* on *M* is ${\mathcal {O}}(\rho ^N)$ if can be written as $\rho ^N{{\tilde{t}}}$ for a tensor field ${{\tilde{t}}}$ that is smooth up to the boundary. Likewise, *t* is ${\mathcal {O}}(\rho ^{-N})$ if $\rho ^Nt$ admits a smooth extension to the boundary. In the obvious way we extend the notation $t_1\sim _N t_2$ with $N>0$ to tensor fields $t_1,t_2$ that are smooth up to the boundary. Observe also that these concepts are compatible in an obvious sense with tensorial operations, like inserting vector fields into metrics, etc.

In this language, a conformally compact metric *g* is $\mathcal O(\rho ^{-2})$ and, writing $g=\rho ^{-2}{{\overline{g}}}$, it satisfies that ${{\overline{g}}}$ is nowhere vanishing and non-degenerate along the boundary. Observe that this implies that the inverse metric $g^{-1}$ is ${\mathcal {O}}(\rho ^2)$ and, in particular, vanishes along $\partial M$. For conformally compact metrics *g* and *h* we can consider the metrics $\rho ^2g$ and $\rho ^2h$ that are smooth up to the boundary and require that $\rho ^2g\sim _N\rho ^2h$, which again is independent of the choice of defining function $\rho$. This defines an equivalence relation on the set of conformally compact metrics, and to simplify notation, we formally write this as $g\sim _{N-2}h$. In the current article we will mainly be interested in the case that $N=n=\dim (M)$ but we carry out most computations for general integers $N>0$, since this does not lead to difficulties and as a preparation for later extensions.

We will start with an equivalence class ${\mathcal {G}}$ of conformally compact metrics on *M* with respect to the equivalence relation $\sim _{N-2}$ for some $N>0$. Observe that for two metrics $g,h\in {\mathcal {G}}$ and a local defining function $\rho$, the metrics $\rho ^2g$ and $\rho ^2h$, by definition, admit a smooth extension to the boundary with the same boundary value. In particular, they induce the same conformal infinity on $\partial M$. Thus the class ${\mathcal {G}}$ of metrics gives rise to a well defined conformal structure on $\partial M$ that we will denote by $[\mathcal G_\infty ]$. As we shall see below, if one metric $g\in {\mathcal {G}}$ is ALH, then the same holds for all metrics in ${\mathcal {G}}$. We shall always assume that this is the case from now on. Let us also remark here that, in the case that $\dim ({\overline{M}})=3$, $[{\mathcal {G}}_\infty ]$ induces a stronger structure that just a conformal structure on $\partial M$ which will be needed in what follows. This will be discussed in more detail below.

From now on, we will sometimes use abstract index notation. In that notation we write $g_{ij}$ for the metric *g*, $g^{ij}$ for its inverse and so on (even though no coordinates or frame field is chosen). Given $g_{ij},h_{ij}\in {\mathcal {G}}$, and fixing a local defining function $\rho$ for $\partial M$, by definition there is a section $\mu _{ij}$ that is smooth up to the boundary such that

2.1 $$\begin{aligned} h_{ij}=g_{ij}+\rho ^{N-2}\mu _{ij}. \end{aligned}$$

Since $g^{ij}$ is ${\mathcal {O}}(\rho ^2)$, we see that $g^{ij}\mu _{ij}=\rho ^2\mu$ for some function $\mu$ that is smooth up to the boundary, whence $g^{ij}(h_{ij}-g_{ij})=\rho ^N\mu$. Using this, we next compute the relation between the defining densities $\sigma ,\tau \in \Gamma ({\mathcal {E}}[1])$ determined by $g_{ij}$ and $h_{ij}$, respectively. Writing

2.2 $$\begin{aligned} h_{ij}=g_{ik}(\delta ^k_j+\rho ^{N-2}g^{k\ell }\mu _{\ell j}), \end{aligned}$$

we can take determinants to find that $\det (h_{ij})=\det (g_{ik})(1+\rho ^N\mu +O(\rho ^{N+1}))\in \Gamma (\mathcal E[-2n])$. (Formally, the determinants are formed by using two copies of the canonical section of $\Lambda ^nTM[-n]$ that expresses the isomorphism between volume forms and densities of conformal weight $-n$ on an oriented manifold. Since two copies of the forms are used, this is well defined even in the non-orientable case, but this will not be needed here.) To obtain $\sigma$ and $\tau$, we have to take $\tfrac{-1}{2n}$th powers, and taking into account that $\sigma$ is ${\mathcal {O}}(\rho )$, we get

2.3 $$\begin{aligned} \tau -\sigma =-\sigma \tfrac{\rho ^N}{2n}\mu +{\mathcal {O}}(\rho ^{N+2}), \end{aligned}$$

so this is ${\mathcal {O}}(\rho ^{N+1})$. Moreover, contracting (2.2) with $g^{ai}$, we get $g^{ai}h_{ij}=\delta ^a_j+\rho ^{N-2}g^{ai}\mu _{ij}$, which in turn easily implies that

2.4 $$\begin{aligned} h^{ij}=g^{ij}-\rho ^{N-2}g^{ik}\mu _{k\ell }g^{\ell j}+\mathcal O(\rho ^{N+3}). \end{aligned}$$

Hence $h^{ij}-g^{ij}$ is ${\mathcal {O}}(\rho ^{N+2})$, which in particular shows that, as claimed above, *h* is ALH provided that *g* has this property.

### Tractors

For a manifold *K* of dimension $n\ge 3$ which is endowed with a conformal structure, the *standard tractor bundle* [1, 5] is a vector bundle $\mathcal TK={\mathcal {E}}^A\rightarrow K$ of rank $n+2$ endowed with the following data.

- A Lorentzian bundle metric, called the *tractor metric*, which we denote by $\langle \, , \rangle$.
- A distinguished isotropic line subbundle ${\mathcal {T}}^1K\subset \mathcal TK$ that is isomorphic to the density bundle ${\mathcal {E}}[-1]$.
- A canonical linear connection, called the *tractor connection*, that preserves the tractor metric and satisfies a non-degeneracy condition.

Since ${\mathcal {T}}^1K$ is isotropic it is contained in $(\mathcal T^1K)^\perp$, and $\langle \, , \rangle$ induces a positive definite bundle metric on $({\mathcal {T}}^1K)^\perp /{\mathcal {T}}^1K$. Via the tractor connection, this quotient gets identified with $\mathcal E^a[-1]$, so the tractor metric gives rise to a section of $\mathcal E_{ab}[2]$, which is exactly the *conformal metric* ${\textbf{g}}_{ab}$ that defines the conformal structure on *K*. These properties together determine the data uniquely up to isomorphism. Observe that the inclusion of $\mathcal T^1K\cong {\mathcal {E}}[-1]$ into $\mathcal TK={\mathcal {E}}^A$ can be viewed as defining a canonical section ${\textbf{X}}^A\in \Gamma ({\mathcal {E}}^A[1])$. Moreover, pairing with ${\textbf{X}}^A$, with respect to the tractor metric, defines an isomorphism $\mathcal TK/({\mathcal {T}}^1K)^\perp \rightarrow {\mathcal {E}}[1]$.

Our strategy for this article requires usage of tractors in a slightly unusual setting. A choice of metric in an equivalence class ${\mathcal {G}}$ as in 2.3 gives us a conformal structure on ${\overline{M}}$ which therefore determines a tractor bundle $\mathcal T{\overline{M}}$. Each of these conformal structures restricts to the same conformal structure $[{\mathcal {G}}_\infty ]$ on $\partial M$, which is thereby canonically associated to ${\mathcal {G}}$. Correspondingly, we have a tractor bundle ${\mathcal {T}}\partial M$ which is canonically associated to ${\mathcal {G}}$. While there is no easy way to relate the tractor bundles on ${\overline{M}}$ coming from different choices of metrics in ${\mathcal {G}}$, we can explicitly identify ${\mathcal {T}}\partial M$ with a subbundle of ${\mathcal {T}}{\overline{M}}$ for any of the choices. Our basic method will be to construct tractor objects on *M* (making choices), prove that they admit a smooth extension to the boundary and that their boundary values lie in the subbundle $\mathcal T\partial M$. Since this bundle is canonical we can then compare the results obtained from different choices.

For our current purposes the “naive” approach to tractors (which avoids the explicit use of Cartan connections or similar tools) is most appropriate and we’ll describe this next. A crucial feature in all approaches to tractors is that the standard tractor bundle admits a simple description depending on the choice of a metric *g* in the conformal class. Such a choice gives an isomorphism $\mathcal E^A\cong {\mathcal {E}}[1]\oplus {\mathcal {E}}_a[1]\oplus {\mathcal {E}}[-1]$, with the last summand corresponding to ${\mathcal {T}}^1K$ and the last two summands corresponding to $({\mathcal {T}}^1K)^\perp$. The resulting elements are usually written as column vectors, with the first component in the top, and there are simple explicit formulae for the tractor metric and the tractor connection in these terms, namely:

2.5 $$\begin{aligned} \left\langle \begin{pmatrix}\sigma \\ \mu _a\\ \nu \end{pmatrix},\begin{pmatrix}{{\tilde{\sigma }}} \\ {{\tilde{\mu }}}_a\\ {{\tilde{\nu }}} \end{pmatrix}\right\rangle =\sigma {{\tilde{\nu }}}+\nu {{\tilde{\sigma }}}+{\textbf{g}}^{ab}\mu _a{{\tilde{\mu }}}_b \end{aligned}$$

with ${\textbf{g}}^{ab}$ denoting the inverse of the conformal metric, and

2.6 $$\begin{aligned} \qquad \nabla ^{{\mathcal {T}}}_a \begin{pmatrix}\sigma \\ \mu _b\\ \nu \end{pmatrix}= \begin{pmatrix}\nabla _a\sigma -\mu _a\\ \nabla _a\mu _b+{\textbf{g}}_{ab}\nu + \textsf {P} _{ab}\sigma \\ \nabla _a\nu -{\textbf{g}}^{ij}\textsf {P} _{ai}\mu _j\end{pmatrix}. \end{aligned}$$

In the right-hand side of this, we use the Levi-Civita connection and the Schouten tensor $\textsf {P} _{ab}$ of *g*. This is a trace-modification of the Ricci tensor $\text {Ric}_{ab}$ of *g* characterized by $\text {Ric}_{ab}=(n-2)\textsf {P} _{ab}+\textsf {P} {\textbf{g}}_{ab}$, where $\textsf {P} : ={\textbf{g}}^{ij}\textsf {P} _{ij}$.

Changing from *g* to a metric ${\widehat{g}}=e^{2f}g$ for $f\in C^\infty (K,{\mathbb {R}})$ there is an explicit formula for the change of the identification in terms of $\Upsilon _a=df$, namely

2.7 $$\begin{aligned} \begin{pmatrix}{\widehat{\sigma }}\\ \widehat{\mu _a}\\ {\widehat{\nu }} \end{pmatrix}=\begin{pmatrix} \sigma \\ \mu _a+\Upsilon _a\sigma \\ \nu -{\textbf{g}}^{ij}(\Upsilon _i\mu _j+\frac{1}{2}\Upsilon _i\Upsilon _j\sigma ) \end{pmatrix}. \end{aligned}$$

Now one may turn around the line of argument and define sections of the tractor bundle as equivalence classes of quadruples consisting of a metric in the conformal class and sections of ${\mathcal {E}}[1]$, ${\mathcal {E}}_a[1]$ and ${\mathcal {E}}[-1]$ with respect to the equivalence relation defined in (2.7). Recall that the behavior of the Schouten tensor under a conformal change is given by

2.8 $$\begin{aligned} {\widehat{\textsf {P} }}_{ab}=\textsf {P} _{ab}-\nabla _a\Upsilon _b+\Upsilon _a\Upsilon _b-\tfrac{1}{2}\Upsilon _i\Upsilon ^ig_{ab}. \end{aligned}$$

Using this, direct computations show that the definitions in (2.5) and (2.6) are independent of the choice of metric, so they give rise to a well-defined bundle metric and linear connection on the resulting bundle. That this cannot be done in individual points is a consequence of the fact that tractors are more complicated geometric objects than tensors, since the action of conformal isometries in a point depends on the two-jet of the isometry in that point. To come to a point-wise construction, one would have to use 1-jets of metrics in the conformal class instead.

In the above discussion we have assumed that $n\ge 3$. Indeed, it is well known that conformal structures in dimension two behave quite differently from higher dimensions. In particular, they do not allow an equivalent description in term of a normal Cartan geometry or of tractors. Still we can obtain boundary tractors as follows. Note first, that associating to a conformal structure a tractor bundle and a tractor metric via formulae (2.7) and (2.5) works without problems in dimension two. This observation is already sufficient for most of our results, where we just need a vector bundle canonically associated to some structure on the boundary. Now one view into the different behavior in dimension two is seen by the fact that the definition of the Schouten tensor $\textsf {P} _{ab}$ via the Ricci curvature breaks down. While there are other ways to understand the Schouten tensor it is nevertheless true that on a 2 dimensional Riemannian manifold there is no natural tensor that transforms conformally according to (2.8). Thus one cannot use (2.6) to associate to a conformal structure a canonical connection on the tractor bundle.

However, in the computations needed to verify that (2.6) leads to a well-defined connection only the transformation law (2.8) for the Schouten tensor under conformal changes is needed, the relation to the Riemann curvature does not play a role. (In fact this computation only involves single covariant derivatives, so there is no chance for curvature terms to arise.) Consequently, the construction of a canonical connection on the tractor bundle extends to dimension two, provided that in addition to a conformal class one associates to each metric in that class a symmetric tensor $\textsf {P} _{ab}$, such that the tensors associated to conformally related metrics satisfy the transformation law (2.8).

The observation just made, for constructing a tractor bundle in dimension 2, is close to the idea of a Möbius structure, but actually it is a slight generalization of the concept of a Möbius structure in the sense of [4]; compare in particular with the MR review [12] of that article. To define a Möbius structure, one requires, in addition, that the trace of the tensor $\textsf {P} _{ab}$ associated to $g_{ab}$ is one half times the scalar curvature of $g_{ab}$. This can be expressed as a normalization condition on the curvature of the tractor connection, but we will not need this. However, in the cases in which we will need the tractor connection in dimension two, we actually will deal with Möbius structures, since we get flat tractor connections.

### Boundary values of tractors

In our usual setting of ${\overline{M}}=M\cup \partial M$, equipped with the conformal class defined by a conformally compact metric, we will deal with standard-tractor-valued differential forms on ${\overline{M}}$. The strategy is to associate to suitable forms, of this type, a boundary value, and interpret this as a form taking values in the standard tractor bundle ${\mathcal {T}}\partial M$ of the conformal infinity. As discussed at the end of Sect. 2.4 above, such a bundle is available in all relevant dimensions $\dim ({\overline{M}})=n\ge 3$. Moreover, since this infinity is the same for all the metrics in a class ${\mathcal {G}}$, as discussed in Sect. 2.2, this allows us to relate the boundary values obtained from different metrics in ${\mathcal {G}}$. However, even for $n\ge 4$, it is not obvious how to relate ${\mathcal {T}}{\overline{M}}$ and ${\mathcal {T}}\partial M$, so we discuss this next. This discussion will also show how to canonically obtain the “abstract Schouten tensors” needed to define a tractor connection on ${\mathcal {T}}\partial M$ for $n=3$. Hence we obtain a uniform description of boundary values in all dimensions.

Provided that one works with metrics that are smooth up to the boundary, the discussion of boundary values of tractors can be reduced to the case of hypersurfaces in conformal manifolds. Observe first that in our setting, it is no problem to relate density bundles on ${\overline{M}}$ and on $\partial M$ of any conformal weight. This is based on the fact that ${\mathcal {E}}[2]$ can always be viewed as the line subbundle spanned by the conformal class and one can form boundary values for metrics in the conformal class (that are smooth up to the boundary). So the densities of weight *w* on $\partial M$ are simply the restriction of the ambient densities of weight *w* i.e. sections of ${\mathcal {E}}[w]|_{\partial M}$.

Now the conformal metric and its inverse define inner products on ${\mathcal {E}}^a[-1]$ and its dual ${\mathcal {E}}_a[1]$. In the case of a boundary, there thus is a unique inward pointing unit normal $n^i\in \Gamma (T{\overline{M}}[-1]|_{\partial M})$ to the subbundle $T\partial M$, and we put $n_i:={\textbf{g}}_{ij}n^j\in \Gamma (T^*{\overline{M}}[1]|_{\partial M})$. We will assume that $n^i$ and $n_i$ are (arbitrarily) extended off the boundary, if needed. For a choice of metric ${\bar{g}}$ (which is smooth up to the boundary) in the conformal class, we observe that the restriction of $\nabla ^{\bar{g}}_in_j$ to $T\partial M\times T\partial M$ is independent of the chosen extension. This is the (weighted) second fundamental form of $\partial M$ in ${\overline{M}}$ with respect to ${{\bar{g}}}$, and we can decompose it into a trace-free part and a trace-part with respect to ${\textbf{g}}_{ab}$. It is well-know that the trace-free part of the second fundamental form is conformally invariant. The trace part can be encoded into the mean curvature $H^{{{\bar{g}}}}\in \Gamma ({\mathcal {E}} [-1])$ of $\partial M$ in ${\overline{M}}$ with respect to ${{\bar{g}}}$. (In our conventions $H^{{{\bar{g}}}}=\frac{1}{n-1}(\nabla _i n^i-n^in^j\nabla _in_j)$.) Its behavior under a conformal change corresponding to $\Upsilon _a$ is given by $H^{\widehat{{{\bar{g}}}}}=H^{{{\bar{g}}}}+\Upsilon _in^i$. It is a classical fact, see Section 2.7 of [1], that these ingredients can be used to construct a conformally invariant *normal tractor*. This provides the standard way to determine whether the boundary value of an interior tractor is a boundary tractor. See also [16] for the proof of the last part.

#### Proposition 2.5

Let ${\overline{M}}=M\cup \partial M$ be a smooth manifold with boundary that is endowed with a conformal structure (which is smooth up to the boundary). Then there is a canonical normal tractor $N^A\in \Gamma (\mathcal TM|_{\partial M})$. If ${{\bar{g}}}$ is a metric in the class that is smooth up to the boundary then, in the splitting corresponding to ${{\bar{g}}}$, $N^A=(0,n_i,-H^{{{\bar{g}}}})$, so $N^AX_A=0$ and for the tractor metric $h_{AB}$ we get $h_{AB}N^AN^B=1$. For $n\ge 4$, the tractor bundle ${\mathcal {T}}\partial M$ with respect to the restriction of the conformal class can be canonically identified with the orthocomplement ${N^A}^\perp \subset {\mathcal {T}}{\overline{M}}|_{\partial M}$.

We next discuss how to make the last statement explicit following [16] and how this extends to the case $n=3$: In a scale ${{\bar{g}}}$ that is smooth up to the boundary, a triple $(\sigma ,\mu _a,\nu )$ is orthogonal to $N^A$ if and only if $\mu _jn^j=H^{{{\bar{g}}}}\sigma$. Such a triple then gets mapped to

$$\begin{aligned} (\sigma ,\mu _a-H^{{{\bar{g}}}} n_a\sigma ,\nu +\tfrac{1}{2}(H^{{{\bar{g}}}})^2\sigma ) \end{aligned}$$

in the splitting corresponding to ${{\bar{g}}}|_{\partial M}$, see Section 6.1 of [11]. In particular, in the case that $\partial M$ is minimal with respect to ${{\bar{g}}}$ (i.e. that $H^{{{\bar{g}}}}$ vanishes identically), we simply get the naïve identification of triples. Given this identification one can compare, along $\partial M$, the ambient tractor connection with that intrinsic to the conformal structure of the boundary in dimensions $n\ge 4$. Although we will not need this fact here, we note that, in particular, the boundary intrinsic tractor connection agrees with the pull-back of the ambient tractor connection if the boundary is totally umbilic and an object called the Fialkow tensor vanishes. Both hold if the structure is locally asymptotically Einstein to a sufficient (low) order. See [11, Theorem 7.4], and its proof.

As mentioned above, these considerations also show how to obtain a tractor connection on ${\mathcal {T}}\partial M$ in the case that $n=3$. We can do this in the setting of hypersurfaces and as discussed in Sect. 2.4, we have to associate an “abstract Schouten tensor” to the metrics in the conformal class $\partial M$. The idea here is simply that for metrics ${{\bar{g}}}$ such that $H^{{\bar{g}}}=0$, we associate the restriction of the Schouten tensor of ${\bar{g}}$ to $\partial M$ as an “abstract Schouten tensor” for the metric ${{\bar{g}}}|_{\partial M}$. If ${{\bar{g}}}$ and $\widehat{{{\bar{g}}}}$ are two such metrics, then for the change $\Upsilon _a$, we get $\Upsilon _in^i=0$, which implies that the restriction of $\Upsilon _i\Upsilon ^i$ to $\partial M$ coincides with the ${\bar{g}}$-trace of the restriction of $\Upsilon _a\Upsilon _b$ to $T\partial M$. From this and the Gauss formula we conclude that restrictions of the Schouten tensors to $\partial M$ satisfy the correct transformation law (2.8). This is already sufficient to obtain a tractor connection on ${\mathcal {T}}\partial M$. Since we know the behavior of all objects under conformal rescalings, one can deduce a description of $\textsf {P} _{ab}$ for general metrics, but we won’t need that here. A different approach to induced Möbius structures on hypersurfaces and more general submanifolds in conformal manifolds can be found in [3].

In any case, it is clear from this description that the above discussion of boundary values now extends to $n=3$. Finally, consider a class ${\mathcal {G}}$, as discussed in Sect. 2.3, with $N\ge 3$ and metrics $g,h\in {\mathcal {G}}$. Then for the conformal classes [*g*] and [*h*] the metrics $\rho ^2g$ and $\rho ^2h$, for a local defining function $\rho$, admit a smooth extension to the boundary. Now by definition $\rho ^2g\sim _N\rho ^2h$. So the difference of their curvatures is ${\mathcal {O}}(\rho ^{N-2})$ and $N-2\ge 1$, which implies that the restrictions of their Schouten tensors to the boundary agree. This implies that, in dimension $n=3$, all metrics in ${\mathcal {G}}$ lead to the same tractor connection on ${\mathcal {T}}\partial M$. In higher dimensions the tractor connection on ${\mathcal {T}}\partial M$ is determined by the conformal structure $[{\mathcal {G}}_\infty ]$, and so the equivalent result holds trivially.

### The scale tractor

We now combine the ideas about boundary tractors with the conformally compact situation. This needs one more basic tool of tractor calculus, the so-called tractor *D*-operator (also called the Thomas *D*-operator). In the simplest situation, which is all that we need here, this is an operator $D^A:{\mathcal {E}}[w]\rightarrow \mathcal E^A[w-1]$, which in triples is defined by

2.9 $$\begin{aligned} D^A\tau =\left( w(n+2w-2)\tau , (n+2w-2)\nabla _a\tau , -{\textbf{g}}^{ij}(\nabla _i\nabla _j+\textsf {P} _{ij})\tau \right) . \end{aligned}$$

Again, a direct computation shows that this is conformally invariant. We will mainly need the case that $w=1$, so that $D^A$ maps sections of the quotient bundle ${\mathcal {E}}[1]$ of $\mathcal E^A$ to sections of ${\mathcal {E}}^A$. Since the first component of $\tfrac{1}{n}D^A\sigma$ is $\sigma$, this operator is referred to as a *splitting operator*. In particular, given a metric in a conformal class, we can apply this splitting operator to the canonical section of ${\mathcal {E}}[1]$ obtained from the volume density of the metric. The resulting section of ${\mathcal {E}}^A$ is called the *scale tractor* associated to the metric.

Computing in the splitting determined by the metric itself, the associated section $\sigma$ of ${\mathcal {E}}[1]$ satisfies $\nabla _a\sigma =0$. Hence, in this splitting, $\tfrac{1}{n}D^A\sigma$ corresponds to $(\sigma ,0,-\tfrac{1}{n}\textsf {P} \sigma )$, where recall $\textsf {P} ={\textbf{g}}^{ij}\textsf {P} _{ij}$. Applying the tractor connection to this, we get $(0,(\textsf {P} _{ab}-\frac{1}{n}{{\textbf {g}}}_{ab}\textsf {P} )\sigma ,-\frac{1}{n}\sigma \nabla _c\textsf {P} )$. Observe that the middle slot of this vanishes iff $\textsf {P} _{ab}$ is pure trace, i.e. iff the metric is Einstein. In that case, $\textsf {P}$ which is just a multiple of the scalar curvature, is constant, hence a metric is Einstein iff its scale tractor is parallel.

Now we move to the case of ${\overline{M}}=M\cup \partial M$ and a conformally compact metric *g* on *M*. By Proposition 2.1, the canonical section $\sigma \in \Gamma ({\mathcal {E}}[1])$ which is parallel for $\nabla ^g$ admits a smooth extension to the boundary (as a defining density). Since we have a conformal structure on all of ${\overline{M}}$ also the scale tractor $I^A:=\tfrac{1}{n}D^A\sigma$ and its covariant derivative $\nabla ^{{\mathcal {T}}}_aI^A$ are smooth up to the boundary. On *M*, we can compute in the splitting determined by *g*, which shows that $\langle I,I\rangle =-\tfrac{2}{n}\sigma ^2\textsf {P} =-\tfrac{2}{n}g^{ij}\textsf {P} _{ij}$. Now the scalar curvature $R=g^{ij}\text {Ric}_{ij}$ of *g* can be written as $2(n-1)\textsf {P}$ and thus $\langle I,I\rangle =-\tfrac{1}{n(n-1)}R$. In particular, if *g* is ALH, then this is identically 1 along the boundary.

Under slightly stronger assumptions, the restriction of $I^A$ to the boundary coincides with the normal tractor $N^A$ from Proposition 2.5. Therefore, $I^A$ can be used to recognize objects that lie in the boundary tractor bundle.

#### Proposition 2.6

Consider a manifold ${\overline{M}}$ with boundary $\partial M$ and interior *M* and a conformally compact metric *g* on *M*; let $\sigma \in \Gamma ({\mathcal {E}}[1])$ be the corresponding density and $I^A:=\tfrac{1}{n}D^A\sigma$ the scale tractor.

If $\langle I,I\rangle =1+{\mathcal {O}}(\rho ^2)$ near to $\partial M$, then the restriction of $I^A$ to $\partial M$ coincides with the normal tractor $N^A$.

#### Proof

See Proposition 7.1 of [11] or Proposition 6 of [15]). $\square$

## The Tractor Mass Cocycle

We consider the tractor version of the classical asymptotically hyperbolic mass here, so the order of asymptotics we need corresponds to $N=n=\dim (M)$ in the notation of Sect. 2.

### The contribution from the trace

Most of the theory we develop applies in the general setting of an oriented manifold ${\overline{M}}$ with boundary $\partial M$, interior *M*, and equipped with a class ${\mathcal {G}}$ of metrics on *M*, as introduced in Sect. 2.3 with $N=n$. The only additional assumption is that the metrics in ${\mathcal {G}}$ are ALH in the sense of Definition 2.2. Given two metrics $g,h\in {\mathcal {G}}$, we denote by $\sigma ,\tau \in \Gamma ({\mathcal {E}}[1])$ the corresponding powers of the volume densities of *g* and *h*. Recall that the class ${\mathcal {G}}$ gives rise to a well-defined standard tractor bundle ${\mathcal {T}}\partial M$ over $\partial M$. Our aim is to associate to *g* and *h* a form $c(g,h)\in \Omega ^{n-1}(\partial M,{\mathcal {T}}\partial M)$, i.e. a top-degree form on $\partial M$ with values in $\mathcal T\partial M$. Further, we want this to satisfy a cocycle property, i.e. that $c(h,g)=-c(g,h)$ and that $c(g,k)=c(g,h)+c(h,k)$ for $g,h,k\in {\mathcal {G}}$.

The first ingredient for this is rather simple: Given $g,h\in {\mathcal {G}}$, we use the conformal structure [*g*] on ${\overline{M}}$, and consider the ${\mathcal {T}}{\overline{M}}$-valued one-form $\tfrac{1}{n}\nabla ^{{\mathcal {T}}}_bD^A(\tau -\sigma )$. We already know that $\tau$ and $\sigma$ admit a smooth extension to the boundary, so this is well defined and smooth up to the boundary. Now on *M*, we can apply the Hodge-$*$-operator determined by *g* to convert this into a ${\mathcal {T}}{\overline{M}}$-valued $(n-1)$-form. The following result shows that this is smooth up to the boundary and that its boundary value is orthogonal to the normal tractor $N^A$ (and non-zero in general). By Proposition 2.5, this boundary value hence defines a form in $\Omega ^{n-1}(\partial M,{\mathcal {T}}\partial M)$.

#### Proposition 3.1

In the setting ${\overline{M}}=M\cup \partial M$ and $g,h\in {\mathcal {G}}$ as described above, let $\rho$ be a local defining function for the boundary. Put ${\overline{g}}:=\rho ^2g$, let ${\overline{\sigma }}\in \Gamma ({\mathcal {E}}[1])$ be the corresponding density and let ${\overline{g}}_{\infty }$ be the boundary value of ${\overline{g}}$.

1. In terms of the canonical section ${{\textbf {X}}}^A\in \mathcal T{\overline{M}}[1]$ from Sect. 2.4 and the function $\mu$ from (2.3) and writing $\rho _a$ for $d\rho$, we get
  3.1 $$\begin{aligned} \nabla ^{{\mathcal {T}}}_bD^A(\tau -\sigma )= \tfrac{n^2-1}{2}\rho ^{n-2}\rho _b\mu {\overline{\sigma }}^{-1}{\textbf{X}}^A+ {\mathcal {O}}(\rho ^{n-1}). \end{aligned}$$
2. The form $\star _g\nabla ^{{\mathcal {T}}}_bD^A(\tau -\sigma )$ is smooth up to the boundary and its boundary value is given by
  3.2 $$\begin{aligned} \tfrac{n^2-1}{2}{\text {vol}}_{{\overline{g}}_{\infty }}\mu _\infty {\overline{\sigma }}_{\infty }^{-1}{\textbf{X}}^A. \end{aligned}$$
  Here ${\text {vol}}_{{\overline{g}}_{\infty }}$ is the volume form of ${\overline{g}}_\infty$, ${\overline{\sigma }}_{\infty }$ is the corresponding 1-density on $\partial M$, and $\mu _{\infty }$ is the boundary value of $\mu$. In particular, this is perpendicular to $N^A|_{\partial M}$ and thus defines an $(n-1)$-form with values in ${\mathcal {T}}\partial M$.

#### Proof

Observe first that for a connection $\nabla$ and a section *s* that both are smooth up to the boundary, and an integer $k>0$, we get $\nabla _a(\rho ^k s)=k\rho ^{k-1}\rho _as+{\mathcal {O}}(\rho ^k)$. This can be applied both to the tractor connection $\nabla ^{{\mathcal {T}}}$ and to the Levi-Civita connection $\nabla$ of ${\overline{g}}$.

Using that ${\overline{\sigma }}=\tfrac{\sigma }{\rho }$, we can write formula (2.3) (still for $N=n$) as $\tau -\sigma =-{\overline{\sigma }}\tfrac{\rho ^{n+1}}{2n}\mu +\mathcal O(\rho ^{n+2})$. Now the defining formula (2.9) for $D^A$ shows that the first two slots of $D^A(\tau -\sigma )$ in the splitting determined by ${\overline{g}}$ are ${\mathcal {O}}(\rho ^{n+1})$ and ${\mathcal {O}}(\rho ^n)$, respectively, while in the last slot the only contribution which is not ${\mathcal {O}}(\rho ^n)$ comes from the double derivative. This shows that

$$\begin{aligned} D^A(\tau -\sigma )=-\tfrac{(n+1)}{2}\rho ^{n-1}{\overline{\sigma }}\mu (- {{\textbf {g}}}^{ij}\rho _i\rho _j){\textbf{X}}^A+{\mathcal {O}}(\rho ^n). \end{aligned}$$

Next, using that ${\overline{g}}^{ij}={\overline{\sigma }}^2{{\textbf {g}}}^{ij}$ and that *g* is ALH, we conclude that ${\overline{\sigma }} {{\textbf {g}}}^{ij}\rho _i\rho _j={\overline{\sigma }}^{-1}+\mathcal O(\rho )$, so we get

$$\begin{aligned} D^A(\tau -\sigma )=\tfrac{n+1}{2}\rho ^{n-1}\mu {\overline{\sigma }}^{-1}{\textbf{X}}^A +{\mathcal {O}}(\rho ^n). \end{aligned}$$

From this (3.1) and hence part (1) follows immediately.

(2) Since ${\overline{M}}$ is oriented we have an isomorphism $\mathcal E[-n]{\mathop {\longrightarrow }\limits ^{\cong }} \Lambda ^nT^*{\overline{M}}$ that can be interpreted as a canonical section $\varvec{\epsilon }_{a_1\dots a_n}\in \Gamma (\Lambda ^nT^*{\overline{M}}[n])$. In terms of this, the volume form of *g* is given by $\sigma ^{-n}\varvec{\epsilon }_{a_1\dots a_n}$. Now by definition, $\star _g\rho ^{n-2}\rho _a$ is given by contracting $\rho ^{n-2}\rho _a$ into the volume form of *g* via $g^{-1}$. So this is given by

3.3 $$\begin{aligned} \sigma ^{-n}\rho ^{n-2}g^{ij}\rho _i\varvec{\epsilon }_{ja_1\dots a_{n-1}}. \end{aligned}$$

Now $\sigma ^{-2}g^{ij}={{\textbf {g}}}^{ij}$, while $\sigma ^{2-n}\rho ^{n-2}={\overline{\sigma }}^{2-n}$. Together with part (1), this shows that

$$\begin{aligned} \star _g\nabla ^{\mathcal {T}}_aD^A(\tau -\sigma )=\tfrac{n^2-1}{2}{\overline{\sigma }}^{2-n}{\textbf{g}}^{ij}\rho _i\varvec{\epsilon }_{ja_1\dots a_{n-1}}\mu {\overline{\sigma }}^{-1}{\textbf{X}}^A+O(\rho ). \end{aligned}$$

This is evidently smooth up to the boundary and its boundary value is a multiple of ${\textbf{X}}^A$ and thus perpendicular to $N^A$ by Proposition 2.5. To obtain the interpretation of the boundary value, we can rewrite (3.3) as ${\overline{\sigma }}^{-n}{\overline{g}}^{ij}\rho _i\varvec{\epsilon }_{ja_1\dots a_{n-1}}$. Since the first and last terms combine to give the volume form of ${\overline{g}}$ and, along the boundary, ${\overline{g}}^{ib}\rho _i$ gives the unit normal with respect to ${\overline{g}}$, we conclude that the boundary value of (3.3) is the volume form of ${\overline{g}}_\infty$. From this, (3.2) and thus part (2) follows immediately. $\square$

There actually is a simpler way to write out the boundary value of $\star _g\nabla ^{{\mathcal {T}}}_bD^A(\tau -\sigma )$ than (3.2) that needs less choices. The function $\mu$ defined in (2.3) of course depends on the choice of the defining function $\rho$, and there is no canonical choice of defining function. However, fixing the metric $g\in {\mathcal {G}}$, we of course get the distinguished defining density $\sigma$, and we can get a more natural version of (2.3) by phrasing things in terms of densities. Namely, for the current setting with $N=n$, we can define $\nu \in \Gamma (\mathcal {E}[-n])$ to be the unique density such that

3.4 $$\begin{aligned} \tau -\sigma =-\tfrac{1}{2n}\sigma ^{n+1}\nu +{\mathcal {O}}(\rho ^{n+2}). \end{aligned}$$

Then of course $\nu$ is uniquely determined by *g* and *h*. In terms of a defining function $\rho$ and the corresponding function $\mu$, we get $\nu =(\tfrac{\rho }{\sigma })^n\mu$, which shows that $\nu$ is smooth up to the boundary and non-zero wherever $\mu$ is non-zero. Let us write the boundary value of $\nu$ as $\nu _\infty$, which by Sect. 2.5 can be interpreted as a density of weight $-n$ on $\partial M$. In the setting of Proposition 3.1, we then have ${\overline{g}}=\rho ^2g$, so ${\overline{\sigma }}=\tfrac{\sigma }{\rho }$. The latter is smooth up to the boundary and from Sect. 2.5 we know that its boundary value is the 1-density ${\overline{\sigma }}_\infty$ on $\partial M$ corresponding to ${\overline{g}}_\infty$. Hence our construction implies that ${\text {vol}}_{{\overline{g}}_\infty }$ corresponds to the density ${{\overline{\sigma }}}_{\infty }^{1-n}$, so (3.2) for the boundary value of $\star _g\nabla ^{{\mathcal {T}}}_bD^A(\tau -\sigma )$ simplifies to

3.5 $$\begin{aligned} \tfrac{n^2-1}{2}{\overline{\sigma }}_\infty ^{-n}\mu _\infty {\textbf{X}}^A= \tfrac{n^2-1}{2}\nu _\infty {\textbf{X}}^A \end{aligned}$$

Using this we can easily prove that we have constructed a cocycle.

#### Corollary 3.2

Let us denote by $c_1(g,h)$ the section of ${\mathcal {T}}\partial M[-n+1]$ associated to $g,h\in {\mathcal {G}}$ via formula (3.2). Then $c_1$ is a cocycle in the sense that $c_1(h,g)=-c_1(g,h)$ and that $c_1(g,k)=c_1(g,h)+c_1(h,k)$ for $g,h,k\in {\mathcal {G}}$.

#### Proof

Expanding $\tau -\sigma =-\tfrac{1}{2n}\sigma ^{n+1}\nu +\mathcal O(\rho ^{n+2})$ as in (3.4) we can compute $c_1(g,h)$ via formula (3.5) from the boundary value of $\nu$. But (3.4) implies $\tau =\sigma +{\mathcal {O}}(\rho ^{n+1})$ and hence $\sigma -\tau =-\tfrac{1}{2n}\tau ^{n+1}(-\nu )+\mathcal O(\rho ^{n+2})$ and hence $c_1(h,g)=-c_1(g,h)$. The second claim follows similarly. $\square$

#### Remark 3.3

The usual classification results for invariant differential operators apply only to irreducible bundles, i.e. natural vector bundles induced by irreducible representations of the conformal group and not to tractor bundles. However, we can use these results to prove that $D^A:\Gamma (\mathcal E[1])\rightarrow \Gamma (\mathcal TK)$ and $\nabla _a^{{\mathcal {T}}}D^A:\Gamma (\mathcal E[1])\rightarrow \Omega ^1(K,\mathcal TK)$ are the unique conformally invariant differential operators with the given source and target. It is known that the only invariant differential operator of low order defined on $\Gamma ({\mathcal {E}}[1])$ with values in an irreducible bundle that is non-zero on conformally flat manifolds is the “conformal–to–Einstein operator” that has values in $S^2_0T^*K[1]$. We’ll discuss this operator in more detail in Sect. 3.9.

Now each quotient of two subsequent components of the filtration

$$\begin{aligned} {\mathcal {T}}^1K\subset ({\mathcal {T}}^1K)^\perp \subset \mathcal TK \end{aligned}$$

from Sect. 2.4 splits into a direct sum of irreducible bundles and $S^2_0T^*K[1]$ is not among these bundles. Now given an invariant differential operator $\Gamma ({\mathcal {E}}[1])\rightarrow \Gamma (\mathcal TK)$, projecting back to sections of $\mathcal TK/(\mathcal T^1K)^\perp \cong {\mathcal {E}}[1]$ one has to obtain a multiple of the identity. Hence subtracting an appropriate multiple of $D^A$, one obtains an operator $\Gamma ({\mathcal {E}}[1])\rightarrow \Gamma ((\mathcal T^1K)^\perp )$. Now the projection of this to $\Gamma ((\mathcal T^1K)^\perp /{\mathcal {T}}^1K)$ has to vanish, whence the values actually have to lie in $\Gamma ({\mathcal {T}}^1K)$. This has to vanish since ${\mathcal {T}}^1K$ is irreducible and not isomorphic to $\mathcal E[1]$ or $S^2_0T^*K[1]$.

Similar arguments apply to $\nabla ^{{\mathcal {T}}}_aD^A$ using the induced filtration of $T^*K\otimes \mathcal TK$, which again leads to quotients that are direct sums of irreducible bundles. This time $S^2_0T^*K[1]\subset T^*K\otimes ({\mathcal {T}}^1K)^\perp /\mathcal T^1K$ is among these bundles, but for all the other bundles obtained in this way it is easy to also see that there are no possible invariant operators coming from $\Gamma ({\mathcal {E}}[1])$ since there is not enough room for curvature terms.

In particular, any invariant differential operator $\Gamma (\mathcal E[1])\rightarrow \Omega ^1(K,\mathcal TK)$ has to have values in $\Gamma (T^*K\otimes ({\mathcal {T}}^1K)^\perp )$ and projecting to $\Gamma (T^*K\otimes ({\mathcal {T}}^1K)^\perp /{\mathcal {T}}^1K)$ has to lead to a multiple of the conformal–to–Einstein operator. In particular, $\nabla ^{{\mathcal {T}}}_aD^A$ induces the conformal–to–Einstein operator in this way. Now if we have given an invariant differential operator $\Gamma (\mathcal E[1])\rightarrow \Omega ^1(K,\mathcal TK)$ we can subtract an appropriate multiple of $\nabla ^{{\mathcal {T}}}_aD^A$ to obtain an invariant operator $\Gamma ({\mathcal {E}}[1])\rightarrow \Gamma (T^*K\otimes \mathcal T^1K)$, which has to vanish by the above considerations.

### The contribution from the trace-free part

We next need another element of tractor calculus that, again, concerns one-forms with values in the standard tractor bundle. Returning to the setting of a general conformal manifold *K*, for $k=1,\dots ,n$, there is a natural bundle map $\partial ^*:\Lambda ^kT^*K\otimes \mathcal TK\rightarrow \Lambda ^{k-1}T^*K\otimes \mathcal TK$, which is traditionally called the *Kostant codifferential*. This has the crucial feature that $\partial ^*\circ \partial ^*=0$, so for each *k*, one obtains nested natural subbundles ${\text {im}}(\partial ^*)\subset \ker (\partial ^*)$ and hence there is the subquotient $\mathcal H_k=\ker (\partial ^*)/{\text {im}}(\partial ^*)$. To make this explicit in low degrees let us write the spaces $\Lambda ^kT^*K\otimes \mathcal TK$ for $k=0,1,2$ in the obvious extension of the vector notation for standard tractors:

$$\begin{aligned} \begin{pmatrix} {\mathcal {E}}[1] \\ {\mathcal {E}}_c[1] \\ {\mathcal {E}}[-1]\end{pmatrix} \overset{\partial ^*}{\longleftarrow } \begin{pmatrix} {\mathcal {E}}_a[1] \\ {\mathcal {E}}_{ac}[1] \\ {\mathcal {E}}_a[-1]\end{pmatrix} \overset{\partial ^*}{\longleftarrow } \begin{pmatrix} {\mathcal {E}}_{[ab]}[1] \\ {\mathcal {E}}_{[ab]c}[1] \\ {\mathcal {E}}_{[ab]}[-1]\end{pmatrix}. \end{aligned}$$

Here ${\mathcal {E}}_{[ab]}$ is the abstract index notation for $\Lambda ^2T^*K$.

The operators $\partial ^*$ are discussed in [7], but what we need here follows from some simple facts and observations, as follows. The maps $\partial ^*$ are conformally invariant bundle maps, so they are induced by linear maps between representations of *CO*(*n*) which are equivariant for the action of the group. Equivariancy under dilations implies that $\partial ^*$ maps each row to the row below, so in particular the bottom row is contained in the kernel of $\partial ^*$. Moreover, Kostant’s version of the Bott-Borel-Weil Theorem implies that ${\mathcal {H}}_0\cong \mathcal E[1]$ and ${\mathcal {H}}_1\cong {\mathcal {E}}_{(ab)_0}[1]$ (symmetric trace-free part). In particular, $\partial ^*:T^*K\otimes \mathcal TK\rightarrow \mathcal TK$ has to map onto the two bottom rows, so ${\text {im}}(\partial ^*)=({\mathcal {T}}^1K)^\perp$. Hence ${\mathcal {H}}_0$ coincides with the natural quotient bundle ${\mathcal {E}}[1]$ of $\mathcal TK$ considered above. This also implies that $\partial ^*$ maps the top slot of $T^*K\otimes \mathcal TK$ isomorphically onto the middle slot of $\mathcal TK$, while its restriction to the middle slot must be a non-zero multiple of the trace. Hence $\ker (\partial ^*)\subset T^*K\otimes \mathcal TK$ consists exactly of those elements for which the top slot vanishes and the middle slot is trace-free.

Similarly, we conclude that $\partial ^*:\Lambda ^2T^*K\otimes \mathcal TK\rightarrow T^*K\otimes \mathcal TK$ has to map the top slot injectively into the middle slot and the middle slot onto the bottom slot of $T^*K\otimes \mathcal TK$. This shows how ${\mathcal {H}}_1\cong \mathcal E_{(ab)_0}[1]$ naturally arises as the subquotient $\ker (\partial ^*)/{\text {im}}(\partial ^*)$ of $T^*K\otimes \mathcal TK$.

Now similarly to the tractor-D operator, the machinery of BGG sequences constructs a conformally invariant *splitting operator*

$$\begin{aligned} S:\Gamma ({\mathcal {H}}_1K)\rightarrow \Gamma (\ker (\partial ^*))\subset \Omega ^1(K,\mathcal TK). \end{aligned}$$

Apart from the fact that for the projection $\pi _H:\ker (\partial ^*)\rightarrow {\mathcal {H}}_1$ one obtains $\pi _H(S(\alpha ))=\alpha$, this operator is characterized by the single property that, for the covariant exterior derivative $d^{\nabla ^{{\mathcal {T}}}}$ induced by the tractor connection, one gets $\partial ^*\circ d^{\nabla ^{{\mathcal {T}}}}(S(\alpha ))=0$ for any $\alpha \in \Gamma ({\mathcal {H}}_1)$ [8]. Similar (but much easier) arguments as in Remark 3.3 show that, up to constant multiples, *S* is the unique invariant differential operator mapping $S^2_0T^*K$ to $\Omega ^1(K,\mathcal TK)$.

To compute the explicit expression for *S*, we again use the notation of triples.

#### Lemma 3.4

Let *K* be a conformal manifold and let *g* be a metric in the conformal class. Then for $\phi =\phi _{ab}\in \Gamma (\mathcal E_{(ab)_0}[1])$, the section $S(\phi )\in \Omega ^1(K,\mathcal TK)$ is, in the splitting determined by *g*, given by

3.6 $$\begin{aligned} (0,\phi _{ab}, \tfrac{-1}{n-1}{\textbf{g}}^{ij}\nabla _i\phi _{aj}). \end{aligned}$$

#### Proof

From above, we know that *S* has values in $\ker (\partial ^*)$ and that this implies that the first component of $S(\phi )$ has to be zero. The fact that $\pi _H\circ S$ is the identity map then shows that the middle component has to coincide with $\phi _{ab}$. Thus it remains to determine the last component, which we temporarily denote by $\psi =\psi _a\in \Gamma ({\mathcal {E}}_a[-1])$. This can be determined by exploiting the fact that $\partial ^*\circ d^{\nabla ^{\mathcal T}}(S(\phi ))=0$. To compute $d^{\nabla ^{\mathcal T}}(0,\phi _{bc},\psi _b)$, we first have to use formula (2.6) to compute $\nabla ^{\mathcal T}_a(0,\phi _{bc},\psi _b)$ viewing the form index *b* as a mere “passenger index”. This leads to $(-\phi _{ba},\nabla _a\phi _{bc}+{\textbf{g}}_{ac}\psi _b,*)$ where we don’t compute the last component, which will not be needed in what follows. Then we have to apply twice the alternation in *a* and *b*, which kills the first component by symmetry of $\phi$ and leads to $2(\nabla _{[a}\phi _{b]c}-\psi _{[a}{\textbf{g}}_{b]c})$ in the middle component. From the description of $\partial ^*$ above we know that $\partial ^*\circ d^{\nabla ^{{\mathcal {T}}}}(S(\phi ))=0$ is equivalent to the fact that this middle component lies in the kernel of a surjective natural bundle map to ${\mathcal {E}}_a[-1]$. By naturality, this map has to be a nonzero multiple of the contraction by ${\textbf{g}}^{bc}$. Using trace-freeness of $\phi$, we conclude that $\partial ^*\circ d^{\nabla ^{{\mathcal {T}}}}(S(\phi ))=0$ is equivalent to

$$\begin{aligned} 0={\textbf{g}}^{ij}(-\nabla _i\phi _{aj})-(n-1)\psi _a, \end{aligned}$$

which gives the claimed formula. $\square$

Now we return to our setting ${\overline{M}}=M\cup \partial M$, and a class ${\mathcal {G}}$ of metrics with $N=n$ as before. Given two metrics $g,h\in {\mathcal {G}}$, we now consider the trace-free part $(h_{ij}-g_{ij})^0$ of $h_{ij}-g_{ij}$ with respect to *g*, which defines a smooth section of ${\mathcal {E}}_{(ab)_0}$. Thus for the density $\sigma \in \Gamma ({\mathcal {E}}[1])$ determined by *g*, we can apply the splitting operator *S* to $\sigma (h_{ij}-g_{ij})^0$, to obtain a ${\mathcal {T}}{\overline{M}}$-valued one-form $\phi _a^B$. We next prove that this has the right asymptotic behavior to apply $\star _g$ and construct a boundary value which lies in $\Omega ^{n-1}(\partial M,\mathcal T\partial M)$, as we did for the trace part in Sect. 3.1 above.

Choosing a local defining function $\rho$ for the boundary, we get the function $\mu _{ij}$ defined in (2.1), and then

3.7 $$\begin{aligned} (h_{ij}-g_{ij})^0=\rho ^{n-2}(\mu _{ij}-\tfrac{1}{n}\rho ^2\mu g_{ij})+{\mathcal {O}}(\rho ^{n-1}), \end{aligned}$$

and clearly $\mu ^0_{ij}:=\mu _{ij}-\tfrac{1}{n}\rho ^2\mu g_{ij}$ defines a section of ${\mathcal {E}}_{(ab)_0}$ that is smooth up to the boundary.

#### Proposition 3.5

In the setting and notation of Proposition 3.1 and using $\mu _{ij}^0$ as defined above, we get:

1. The form $S(\sigma (h_{ij}-g_{ij})^0)\in {\mathcal {E}}_a^A$ is given by
  3.8 $$\begin{aligned} -\rho ^{n-2}{\overline{g}}^{ij}\rho _i\mu ^0_{aj}{\overline{\sigma }}^{-1}{\textbf{X}}^A+\mathcal O(\rho ^{n-1}). \end{aligned}$$
2. The form $\star _gS(\sigma (h_{ij}-g_{ij})^0)$ is smooth up to the boundary and its boundary value is given by
  3.9 $$\begin{aligned} -{\overline{g}}^{ij}{\overline{g}}^{k\ell }\rho _i\rho _k\mu ^0_{j\ell }{\text {vol}}_{{\overline{g}}_\infty } {\overline{\sigma }}_{\infty }^{-1}{\textbf{X}}^A. \end{aligned}$$
  This is perpendicular to $N^A|_{\partial M}$ and thus by Proposition 2.5 defines a form in $\Omega ^{n-1}(\partial M,\mathcal T\partial M)$.

#### Proof

1. As before, we will work in the splitting determined by ${\overline{g}}_{ij}$ throughout the proof. By construction $\sigma (h_{ij}-g_{ij})^0=\sigma \rho ^{n-2}\mu _{ij}^0={\overline{\sigma }}\rho ^{n-1}\mu _{ij}^0$ is ${\mathcal {O}}(\rho ^{n-1})$. Using Lemma 3.4, we see that the first slot of $S(\sigma (h_{ij}-g_{ij})^0)$ vanishes and its middle slot is ${\mathcal {O}}(\rho ^{n-1})$. Using the observation from the beginning of the proof of Proposition 3.1 we see that the covariant derivative of ${\overline{\sigma }}\rho ^{n-1}\mu _{ij}^0$, with respect to the Levi-Civita connection of ${\overline{g}}_{ij}$, is given by $(n-1){\overline{\sigma }}\rho ^{n-2}\rho _k\mu _{ij}^0+\mathcal O(\rho ^{n-1})$. Using this, the claimed formula follows immediately from Lemma 3.4 and the fact that ${\overline{g}}^{ij}={\overline{\sigma }}^2{\textbf{g}}^{ij}$.
2. Proceeding as in the proof of Proposition 3.1, we now show that $\star _gS((h_{ij}-g_{ij})^0)$ is given by
  $$\begin{aligned} -{\overline{g}}^{ij}\rho _i\mu ^0_{kj}{\overline{g}}^{k\ell }\varvec{\epsilon }_{\ell a_1\dots a_{n-1}}{\overline{\sigma }}^{-n-1}{\textbf{X}}^A. \end{aligned}$$
  This is evidently smooth up to the boundary and, as observed there, ${\overline{\sigma }}^{-n}\varvec{\epsilon }_{a_1\dots a_n}$ is the volume form of ${\overline{g}}_{ij}$. Writing ${\text {vol}}_{{\overline{g}}}|_{\partial M}$ as $d\rho \wedge {\text {vol}}_{{\overline{g}}_\infty }$ and using that the image of $d\rho$ in $\Omega ^1(\partial M)$ vanishes, we directly get the claimed formula for the boundary value. The final statement follows the same argument as in Proposition 3.1. $\square$

Similarly as in Sect. 3.1 above, this admits a more natural interpretation when working with densities. Again fixing $N=n$, instead of (2.1) we can start from

3.10 $$\begin{aligned} h_{ij}=g_{ij}+\sigma ^{n-2}\nu _{ij}, \end{aligned}$$

where $\nu _{ij}\in \Gamma ({\mathcal {E}}_{(ij)}[-n+2])$ now is a weighted symmetric two-tensor that is smooth up to the boundary. For a choice of local defining function $\rho$, the relation to (2.1) is described by $\nu _{ij}=(\frac{\rho }{\sigma })^{n-2}\mu _{ij}$. This immediately implies that

$$\begin{aligned} {\textbf{g}}^{ij}\nu _{ij}=(\tfrac{\rho }{\sigma })^{n-2}\tfrac{1}{\sigma ^2}g^{ij}\mu _{ij}=(\tfrac{\rho }{\sigma })^n\mu =\nu , \end{aligned}$$

where $\nu \in \Gamma ({\mathcal {E}}[-n])$ is the density used in Sect. 3.1. The tracefree part $\nu ^0_{ij}$, then of course is $\nu _{ij}-\tfrac{1}{n} {\textbf{g}}_{ij}\nu =(\frac{\rho }{\sigma })^{n-2}\mu ^0_{ij}$. On the other hand, the fact that $g_{ij}$ is ALH shows that $\tfrac{1}{\rho ^2}g^{ij}\rho _i\rho _j$ is identically one along the boundary. Using $g^{ij}=\sigma ^2{\textbf{g}}^{ij}$, we conclude that $\tfrac{\sigma }{\rho }{\textbf{g}}^{ij}\rho _i\in {\mathcal {E}}^a[-1]$ coincides, along $\partial M$, with the conformal unit normal $n^j$ from Sect. 2.5. Hence ${\overline{g}}^{ij}\rho _j={\overline{\sigma }}n^i$ and hence we can rewrite formula (3.8) as $-\sigma ^{n-2}\nu ^0_{ij}n^j{\textbf{X}}^A+{\mathcal {O}}(\rho ^{n-1})$, where we have extended $n^j$ arbitrarily off the boundary.

To rewrite the formula (3.9) for the boundary value in a similar way, we use the observation that ${\text {vol}}_{{\overline{g}}_\infty }$ corresponds to $(\tfrac{\rho }{\sigma })^{n-1}$, as discussed in Sect. 3.1. Using this and the above, see that (3.9) equals

3.11 $$\begin{aligned} -n^in^j(\nu ^0_{ij})_\infty {\textbf{X}}^A, \end{aligned}$$

where $(\nu ^0_{ij})_\infty$ indicates the boundary value of $\nu ^0_{ij}$. Using this formulation, it is easy to prove that we obtain another cocycle.

#### Corollary 3.6

Let us denote the section of ${\mathcal {T}}\partial M[-n+1]$ associated to $g,h\in {\mathcal {G}}$ via formula (3.9) by $c_2(g,h)$. Then $c_2$ is a cocycle in the sense that $c_2(h,g)=-c_2(g,h)$ and that $c_2(g,k)=c_2(g,h)+c_2(h,k)$ for $g,h,k\in {\mathcal {G}}$.

#### Proof

Suppose that the tracefree part of $h_{ij}-g_{ij}$ with respect to *g* is given by $\sigma ^{n-2}\nu ^0_{ij}$ for $\nu ^0_{ij}\in \Gamma (\mathcal E_{ij}[2-n])$ and similarly the tracefree part of $(g_{ij}-h_{ij})^0$ with respect to *h* corresponds to ${{\tilde{\nu }}}^0_{ij}$. Then one immediately verifies that ${{\tilde{\nu }}}^0_{ij}=-\nu ^0_{ij}+{\mathcal {O}}(\rho ^{n-2})$, and thus using formula (3.11) to compute boundary values readily implies $c_2(h,g)=-c_2(g,h)$. The second claim follows similarly. $\square$

### Diffeomorphisms

We next start to study the compatibility of the cocycles we have constructed above with diffeomorphisms. There are various concepts of compatibility here, and we have to discuss some background first. Recall that a diffeomorphism $\Psi :{\overline{M}}\rightarrow {\overline{M}}$ of a manifold with boundary maps *M* to *M* and $\partial M$ to $\partial M$. This also shows that for $x\in \partial M$, the linear isomorphism $T_x\Psi :T_xM\rightarrow T_{\Psi (x)}M$ maps the subspace $T_x\partial M$ to $T_{\Psi (x)}\partial M$. It follows that for a defining function $\rho$ for $\partial M$, also $\Psi ^*\rho =\rho \circ \Psi$ is a defining function for $\partial M$. This also works for a local defining function defined on a neighborhood of $\Psi (x)$, which gives rise to a local defining function defined on a neighborhood of *x*.

These results imply that the relation $\sim _N$ on tensor fields defined in Sect. 2.3 is compatible with diffeomorphisms in the sense that $t\sim _N{{\tilde{t}}}$ implies $\Psi ^*t\sim _N\Psi ^*{{\tilde{t}}}$ for each $N>0$. In particular, given an equivalence class ${\mathcal {G}}$ of conformally compact metrics, also $\Psi ^*{\mathcal {G}}$ is such an equivalence class. We are particularly interested in the case that $\Psi ^*\mathcal G={\mathcal {G}}$, in which we say that $\Psi$ *preserves* ${\mathcal {G}}$. The diffeomorphisms with this property clearly form a subgroup of the diffeomorphism group ${\text {Diff}}({\overline{M}})$ which we denote by ${\text {Diff}}_{{\mathcal {G}}}({\overline{M}})$. From our considerations it follows immediately that this is equivalent to the fact that there is one metric $g\in {\mathcal {G}}$ such that $\Psi ^*g\in {\mathcal {G}}$ or equivalently $\Psi ^*g\sim _{N-2}g$. (In [10] an analogous property is phrased by saying that $\Psi$ is an “asymptotic isometry” of *g*. We don’t use this terminology since $\Psi$ is not better compatible with *g* than with any other metric in ${\mathcal {G}}$.)

Recall from Sect. 2.3 that all metrics in ${\mathcal {G}}$ give rise to the same conformal infinity on $\partial M$. This implies that for $\Psi \in {\text {Diff}}_{{\mathcal {G}}}({\overline{M}})$ the restriction $\Psi _\infty :=\Psi |_{\partial M}$ is not only a diffeomorphism, but actually a conformal isometry of the conformal infinity of $\mathcal G$. In particular, it induces a well-defined bundle automorphism on the standard tractor bundle ${\mathcal {T}}\partial M$ and hence we can pull back sections of ${\mathcal {T}}\partial M$ along $\Psi _\infty$. This also works for $n=3$ without problems. Using this, we can prove the first and simpler compatibility condition of our cocycles with diffeomorphisms.

#### Proposition 3.7

Consider a manifold ${\overline{M}}=M\cup \partial M$ with boundary and a class ${\mathcal {G}}$ of metrics, in the case $N=n$. Then the cocycles constructed in Propositions 3.1 and 3.5 are compatible with the action of a diffeomorphism $\Psi \in {\text {Diff}}_{{\mathcal {G}}}({\overline{M}})$ in the sense that for each such cocycle $c(\Psi ^*g,\Psi ^*h)=(\Psi _\infty )^*c(g,h)$. Here $\Psi _\infty =\Psi |_{\partial M}$, and on the right hand side we have the action of a conformal isometry of the conformal infinity of ${\mathcal {G}}$ (on $\partial M$) on tractor-valued forms.

#### Proof

This basically is a direct consequence of the invariance properties of the constructions we use. If *g* corresponds to $\sigma \in \Gamma ({\mathcal {E}}[1])$, then of course $\Psi ^*g$ corresponds to $\Psi ^*\sigma$. Moreover, $\Psi _\infty$ defines a conformal isometry between the conformal structures on $\partial M$ induced by $\Psi ^*g$ and *g*, respectively. Similarly, $\Psi ^*h$ corresponds to $\Psi ^*\tau$ and naturality of the tractor constructions implies that $\nabla ^{{\mathcal {T}}}_aD_B(\Psi ^*\tau -\Psi ^*\sigma )$ (computed in the conformal structure $[\Psi ^*g]$) equals $\Psi ^*(\nabla ^{\mathcal T}_aD_B(\tau -\sigma ))$. Since $\Psi |_M$ is an isometry from $\Psi ^*g$ to *g*, we get $\Psi ^*{\text {vol}}_g={\text {vol}}_{\Psi ^*g}$, which implies compatibility with the Hodge-star. Hence on *M*, we get

$$\begin{aligned} \star _{\Psi ^*g}\nabla ^{\mathcal T}_aD_B(\Psi ^*\tau -\Psi ^*\sigma )=\Psi ^*(\star _g\nabla ^{\mathcal T}_aD_B(\tau -\sigma )) \end{aligned}$$

and since both sides admit a smooth extension to the boundary, the boundary values have to coincide, too. But these than are exactly $c_1(\Psi ^*g,\Psi ^*h)$ and the pull back induced by the conformal isometry $\Psi _\infty$ of $c_1(g,h)$. This completes the proof for $c_1$.

For $c_2$, we readily get that $(\Psi ^*h-\Psi ^*g)^0$ (tracefree part with respect to $\Psi ^*g$) coincides with $\Psi ^*(h-g)^0$ (tracefree part with respect to *g*). Using naturality of the splitting operator *S*, the proof is completed in the same way as for $c_1$. $\square$

### Diffeomorphisms asymptotic to the identity

To move towards a more subtle form of compatibility of our cocycles with diffeomorphisms, we need a concept of asymptotic relation between diffeomorphisms.

#### Definition 3.8

Let ${\overline{M}}=M\cup \partial M$ be a manifold with boundary and let $\Psi ,{{\tilde{\Psi }}}:{\overline{M}}\rightarrow {\overline{M}}$ be diffeomorphisms.

1. We say that $\Psi$ and ${\widetilde{\Psi }}$ are asymptotic of order $N>0$ and write $\Psi \sim _N{\widetilde{\Psi }}$ if and only if for any function $f\in C^\infty ({\overline{M}}, {\mathbb {R}})$ we get $f\circ \Psi \sim _Nf\circ {\widetilde{\Psi }}$ in the sense of Sect. 2.3.
2. For $N>0$, we define ${\text {Diff}}_0^N({\overline{M}})$ to be the set of diffeomorphisms which are asymptotic to the identity ${\text {id}}_{{\overline{M}}}$ of order *N*.

Since $\sim _N$ clearly defines an equivalence relation on functions, we readily see that it is an equivalence relation on diffeomorphisms. Moreover, since the pull back of a local defining function for $\partial M$ along a diffeomorphism of ${\overline{M}}$ again is a local defining function, we conclude that $\Psi \sim _N{\widetilde{\Psi }}$ implies $\Psi \circ \Phi \sim _N{\widetilde{\Psi }}\circ \Phi$ and $\Phi \circ \Psi \sim _N\Phi \circ {\widetilde{\Psi }}$ for any diffeomorphism $\Phi$ of ${\overline{M}}$. In particular, this shows either of ${\widetilde{\Psi }}^{-1}\circ \Psi \sim _N{\text {id}}$ and ${\widetilde{\Psi }}\circ \Psi ^{-1}\sim _N{\text {id}}$ is equivalent to $\Psi \sim _N{\widetilde{\Psi }}$.

On the other hand, we need some observations on charts. Given a manifold ${\overline{M}}=M\cup \partial M$ with boundary, take a point $x\in \partial M$. Then by definition, there is a chart (*U*, *u*) around *x*, so *U* is an open neighborhood of *x* in ${\overline{M}}$ and $u:U\rightarrow u(U)$ is a diffeomorphism onto an open subset of an *n*-dimensional half space. Then *u* restricts to a diffeomorphism between the open neighborhood $U\cap \partial M$ of *x* in $\partial M$ and the open subspace $u(U)\cap ({\mathbb {R}}^{n-1}\times \{0\})$ of ${\mathbb {R}}^{n-1}$. Then by definition, the last coordinate function $u^n$ is a local defining function of $\partial M$. Conversely, any local defining function can locally be used as such a coordinate function in a chart.

If $\Psi \in {\text {Diff}}({\overline{M}})$ is a diffeomorphism, then for a chart (*U*, *u*) also $(\Psi ^{-1}(U),u\circ \Psi )$ is a chart. If ${\widetilde{\Psi }}$ is another diffeomorphism such that ${\widetilde{\Psi }}|_{\partial M}=\Psi |_{\partial M}$, then $V:=\Psi ^{-1}(U)\cap {\widetilde{\Psi }}^{-1}(U)$ is an open subset in ${\overline{M}}$ which contains $\Psi ^{-1}(U\cap \partial M)$. For any tensor field *t* defined on *U*, both $\Psi ^*t$ and ${\widetilde{\Psi }}^*t$ are defined on *V*, and can be compared asymptotically there. Using these observations, we start by proving a technical lemma.

#### Lemma 3.9

Let ${\overline{M}}=M\cup \partial M$ be a smooth manifold with boundary, let $\Psi ,{\widetilde{\Psi }}\in {\text {Diff}}({\overline{M}})$ be diffeomorphisms, and fix $N>0$. Then the following conditions are equivalent:

(i) $$\Psi \sim _{N+1}{\widetilde{\Psi }}$$
(ii) $\Psi |_{\partial M}={\widetilde{\Psi }}|_{\partial M}$ and for any tensor field *t* on ${\overline{M}}$ (including functions), we get $\Psi ^*t\sim _N{\widetilde{\Psi }}^*t$.
(iii) $\Psi |_{\partial M}={\widetilde{\Psi }}|_{\partial M}$ and for each $x\in \partial M$, there is an chart (*U*, *u*) for ${\overline{M}}$, with $x\in U$, whose coordinate functions $u^i$ satisfy $\Psi ^*u^i\sim _{N+1}{\widetilde{\Psi }}^*u^i$ locally around $\Psi ^{-1}(x)$.

#### Proof

Replacing $\Psi$ by ${\widetilde{\Psi }}^{-1}\circ \Psi$ we may without loss of generality assume that ${\widetilde{\Psi }}={\text {id}}_{{\overline{M}}}$, which we do throughout the proof.

(i)$\Rightarrow$(iii): We first claim that $\Psi |_{\partial M}={\text {id}}_{\partial M}$. For $x\in \partial M$, take an open neighborhood *W* of *x* in $\partial M$. Then there is a bump function $f\in C^\infty ({\overline{M}},{\mathbb {R}})$ with values in [0, 1] such that $f(x)=1$ and such that $f^{-1}(\{1\})\cap \partial M\subset W$. By assumption $f\circ \Psi \sim _{N+1}f$, so in particular, these functions have to agree on $\partial M$ and hence at *x*. Since $\Psi (x)\in \partial M$, by construction, we get $\Psi (x)\in W$. Since *W* was arbitrary, this implies that $\Psi (x)=x$ and hence the claim. Having this at hand, we take any chart (*U*, *u*) with $x\in U$, extend the coordinate functions $u^i$ to globally defined functions on *M* without changing them locally around *x*, and then (i) immediately implies that $\Psi ^*u^i\sim _{N+1}u^i$ locally around *x*.

(iii)$\Rightarrow$(ii): For any tensor field *t*, it suffices to verify $\Psi ^*t\sim _N t$ locally around each boundary point $x\in \partial M$. Fixing *x*, we take a chart (*U*, *u*) as in (iii) and its coordinate functions $u^i$ and we work on $V=\Psi ^{-1}(U)\cap U$. Taking a vector field $\xi \in {\mathfrak {X}}(U)$ we can compare $\Psi ^*\xi$ and $\xi$ on *V*. We can do this via coordinate expressions with respect to the chart (*U*, *u*) and we denote by $\xi ^i$ and $(\Psi ^*\xi )^i$ the component functions. By assumption, $u^i\circ \Psi =u^i+{\mathcal {O}}(\rho ^{N+1})$ and differentiating this with $\Psi ^*\xi$, we obtain $(\Psi ^*\xi ) (u^i\circ \Psi )=(\Psi ^*\xi )(u^i)+{\mathcal {O}}(\rho ^N)$. Thus we conclude that $(\Psi ^*\xi )(u^i\circ \Psi )\sim _N(\Psi ^*\xi )^i$. But by definition of the pull back, we get $(\Psi ^*\xi )(u^i\circ \Psi )=\xi (u^i)\circ \Psi \sim _{N+1}\xi ^i$. Overall, we conclude that $(\Psi ^*\xi )^i\sim _N\xi ^i$ on *V*, which implies that $\Psi ^*\xi \sim _N\xi$ on *V* and hence we get condition (ii) for vector fields.

In particular, this implies that the coordinate vector fields $\partial _i$ of the chart (*U*, *u*) satisfy $\Psi ^*\partial _i\sim _N\partial _i$ on *V*. On the other hand, applying the exterior derivative to $u^i\circ \Psi \sim _{N+1}u^i$, we conclude that $\Psi ^*du^i=d(u^i\circ \Psi )\sim _Ndu^i$. Of course, on *V* the $du^i$ coincide with the coordinate one-forms of the chart (*U*, *u*). Now given a tensor field *t* of any type, we can take $\Psi ^*t$ and hook in vector fields $\Psi ^*\partial _{i_a}$ and one-forms $\Psi ^*du^{j_b}$. On *V* this by construction produces one of the component functions of *t* up to ${\mathcal {O}}(\rho ^N)$. On the other hand, by definition of the pull back, this coincides with the composition of the corresponding coordinate function of *t* with $\Psi$, and hence with that coordinate function up to $\mathcal O(\rho ^{N+1})$, so (ii) is satisfied in general.

(ii)$\Rightarrow$(i): Let $f\in C^\infty ({\overline{M}},{\mathbb {R}})$ be a smooth function. Then by assumption we know that $f\circ \Psi \sim _Nf$, so choosing a local defining function $\rho$ for $\partial M$, we get $f\circ \Psi =f+\rho ^N{{\tilde{f}}}$ for some smooth function ${{\tilde{f}}}\in C^\infty ({\overline{M}},{\mathbb {R}})$. But then $\Psi ^*df=d(f\circ \Psi )=df+N\rho ^{N-1}{{\tilde{f}}}d\rho +\mathcal O(\rho ^N)$. However, condition (ii) also says that $\Psi ^*df\sim _Ndf$ and since $d\rho |_{\partial M}$ is nowhere vanishing, this implies that ${{\tilde{f}}}|_{\partial M}=0$. But this implies that $f\circ \Psi \sim _{N+1}f$ and hence condition (i) follows. $\square$

### The relation to adapted defining functions

In a special case and in quite different language, it has been observed in [10] that there is a close relation between diffeomorphisms asymptotic to the identity and adapted defining functions. We start discussing this with the following lemma.

#### Lemma 3.10

In our usual setting, of ${\overline{M}}=M\cup \partial M$, let ${\mathcal {G}}$ be an equivalence class of ALH metrics on *M* for the relation $\sim _{N-2}$ for some $N\ge 3$. Take two metrics $g,h\in \mathcal G$, and let $\rho$ and *r* be local defining functions for $\partial M$ defined on the same open subset $U\subset {\overline{M}}$. If $\rho$ is adapted to *g* and *r* is adapted to *h* in the sense of Definition 2.2 and if $\rho ^2g_{ij}$ and $r^2h_{ij}$ induce the same metric on the boundary, then $\rho \sim _{N+1}r$.

#### Proof

We have to analyze the asymptotics of solutions to the PDE that governs the change to an adapted defining function. Replacing ${\overline{M}}$ by an appropriate open subset, we may assume that $\rho$ and *r* are defined on all of ${\overline{M}}$. Then we can write $r=\rho e^v$ for some smooth function $v\in C^\infty ({\overline{M}},{\mathbb {R}})$ which gives $dr=rdv+e^vd\rho$. In abstract index notation, this reads as $r_i=rv_i+e^v\rho _i$. The fact that *r* is adapted to $h_{ij}$ says that $r^{-2}h^{ij}r_ir_j$ is identically 1 on a neighborhood of the boundary, and inserting we conclude that

3.12 $$\begin{aligned} 1\equiv \rho ^{-2}h^{ij}\rho _i\rho _j+2\rho ^{-1}h^{ij}\rho _iv_j+h^{ij}v_iv_j. \end{aligned}$$

Observe that $\rho ^{-2}h^{ij}$, $\rho _i$ and $v_i$ are all smooth up to the boundary, so the terms in the right hand side are $\mathcal O(1)$, ${\mathcal {O}}(\rho )$, and ${\mathcal {O}}(\rho ^2)$, respectively.

Now on the one hand, since $g,h\in {\mathcal {G}}$, we know from (2.4) that $\rho ^{-2}h^{ij}=\rho ^{-2}g^{ij}+\mathcal O(\rho ^N)$. Since $\rho$ is adapted to *g*, this means that the first term in the right hand side of (3.12) is $1+\mathcal O(\rho ^N)$. Inserting into (3.12), we conclude that

3.13 $$\begin{aligned} 2\rho ^{-1}h^{ij}\rho _iv_j+h^{ij}v_iv_j={\mathcal {O}}(\rho ^N). \end{aligned}$$

On the other hand, $r^2h_{ij}=e^{2v}\rho ^2h_{ij}$, and $r^2h_{ij}$, by assumption, is smooth up to the boundary with the same boundary value as $\rho ^2g_{ij}$. Hence our assumption on the induced metrics on the boundary imply that $e^{2v}|_{\partial M}=1$, so *v* has to vanish identically along the boundary and hence $v=\mathcal O(\rho )$. Inductively, putting $v=\rho ^\ell {{\tilde{v}}}$ for $\ell \ge 1$, we get $v_i=\ell \rho ^{\ell -1}{{\tilde{v}}}\rho _i+{\mathcal {O}}(\rho ^\ell )$, which implies that the left hand side of (3.13) becomes $2\ell \rho ^\ell {\tilde{v}}\rho ^{-2}h^{ij}\rho _i\rho _j+\mathcal O(\rho ^{\ell +1})$. As long as $\ell <N$, this shows that ${{\tilde{v}}}= {\mathcal {O}} (\rho )$, so we conclude that we can write $v=\rho ^N{{\tilde{v}}}$ where ${{\tilde{v}}}$ is smooth up to the boundary. But then

$$\begin{aligned} r=\rho e^v=\rho (1+\rho ^N{{\tilde{v}}}+\mathcal O(\rho ^{N+1}))=\rho +{\mathcal {O}}(\rho ^{N+1}), \end{aligned}$$

which completes the proof. $\square$

Using this, we can now establish several important properties of diffeomorphisms that are asymptotic to the identity.

#### Theorem 3.11

Let ${\overline{M}}=M\cup \partial M$ be a smooth manifold with boundary and, for some $N\ge 3$, let ${\mathcal {G}}$ be an equivalence class of ALH metrics on *M* for the relation $\sim _{N-2}$. Let us denote by $[{\mathcal {G}}_\infty ]$ the conformal structure on $\partial M$ defined by the conformal infinity of ${\mathcal {G}}$ and by ${\text {Conf}}(\partial M,[{\mathcal {G}}_\infty ])$ the group of its conformal isometries. Then we have

1. ${\text {Diff}}^{N+1}_0({\overline{M}})$ is a normal subgroup in ${\text {Diff}}({\overline{M}})$ and is contained in ${\text {Diff}}_{{\mathcal {G}}}({\overline{M}})$.
2. Restriction of diffeomorphisms to the boundary induces a homomorphism ${\text {Diff}}_{{\mathcal {G}}}({\overline{M}})\rightarrow {\text {Conf}}(\partial M,[\mathcal G_\infty ])$ with kernel ${\text {Diff}}^{N+1}_0({\overline{M}})$.

#### Proof

1. The observations on the relation $\sim _N$ for diffeomorphisms we have made after Definition 3.8 readily imply that ${\text {Diff}}^{N+1}_0({\overline{M}})$ is stable under inversions as well as compositions, and conjugations by arbitrary elements of ${\text {Diff}}({\overline{M}})$. Hence ${\text {Diff}}^{N+1}_0({\overline{M}})$ is a normal subgroup of ${\text {Diff}}({\overline{M}})$. Taking $g\in {\mathcal {G}}$ and a local defining function $\rho$ for $\partial M$, we know that $\rho ^2g_{ij}$ admits a smooth extension to the boundary. Thus, given $\Psi \in {\text {Diff}}^{N+1}_0({\overline{M}})$, we may apply part (ii) of Lemma 3.9 to conclude that $\Psi ^*(\rho ^2g_{ij})\sim _N\rho ^2g_{ij}$. Now $\Psi ^*(\rho ^2)=(\rho \circ \Psi )^2$ and $\rho \circ \Psi \sim _{N+1}\rho$. Hence $\rho ^2\Psi ^*g_{ij}\sim _N \Psi ^*(\rho ^2g_{ij})\sim _N\rho ^2g_{ij}$ and restricting to *M* we conclude that $\Psi ^*g_{ij}\in {\mathcal {G}}$. This completes the proof of (1).
2. It follows readily from the definitions that, for $\Psi \in {\text {Diff}}_{{\mathcal {G}}}({\overline{M}})$, the diffeomorphism $\Psi |_{\partial M}$ of $\partial M$ preserves the conformal structure $[{\mathcal {G}}_\infty ]$. Thus we get a homomorphism ${\text {Diff}}_{{\mathcal {G}}}({\overline{M}})\rightarrow {\text {Conf}}(\partial M,[{\mathcal {G}}_\infty ])$ as claimed and it remains to prove the claim about the kernel. So let us take a diffeomorphism $\Psi \in {\text {Diff}}_{{\mathcal {G}}}({\overline{M}})$ such that $\Psi |_{\partial M}={\text {id}}_{\partial M}$ and we want to show that $\Psi \sim _{N+1}{\text {id}}$. To prove this, we can apply condition (iii) of Lemma 3.9 and work locally around a boundary point *x*. Let us choose $g\in {\mathcal {G}}$ and a local defining function $\rho$ for $\partial M$ which is adapted to *g* and defined on some open neighborhood *U* of *x* in ${\overline{M}}$. Now we consider the normal field (to $\rho$ level sets) determined by *g* and $\rho$, i.e. we put $\xi ^i:=\rho ^{-2}g^{ij}\rho _j$. This admits a smooth extension to the boundary, and the fact that $\rho$ is adapted to *g* exactly says that $\rho ^2g_{ij}\xi ^i\xi ^j$ is identically one on a neighborhood of the boundary. For $x\in \partial M$, we now work on an open neighborhood *W* of *x* in ${\overline{M}}$ such that $W\subset \Psi ^{-1}(U)\cap U$. On *W*, we can pull back all our data by $\Psi$, thus obtaining $h_{ij}:=\Psi ^*g_{ij}$, $r:=\rho \circ \Psi$, and $\eta :=\Psi ^*\xi$. By assumption, $h_{ij}\in {\mathcal {G}}$ and pulling back the defining equation for $\xi ^i$ we get $\eta ^i=r^{-2}h^{ij}r_j$. Also by pulling back, we readily see that the $r^2h_{ij}\eta ^i\eta ^j$ is identically one on a neighborhood of the boundary. Hence we conclude that the local defining function *r* is adapted to $h_{ij}$ and hence Lemma 3.10 shows that $r\sim _{N+1}\rho$. Observe that this implies that $r_j\sim _N\rho _j$ and together with $g^{ij}\sim _{N+2}h^{ij}$ the defining equations for $\xi$ and $\eta$ show that $\xi \sim _N\eta$. Next, we pass to an appropriate collar of the boundary. We can choose an open neighborhood *V* of *x* in $\partial M$ and a positive number $\epsilon$ such that the flow map $(y,t)\mapsto {\text {Fl}}^\eta _t(y)$ defines a diffeomorphism $\phi$ from $V\times [0,\epsilon )$ onto an open subset contained in $\Psi ^{-1}(W)\cap W$, and on which $\rho$ satisfies $(\rho ^2 g)^{-1}(d\rho ,d\rho )=1$. Now let us define $\partial _t:=\phi ^*\eta$. Note that this is the coordinate vector field for any product chart on $V\times [0,\epsilon )$ induced by some chart on *V*. Since $\eta =\Psi ^*\xi$, the fact that $\Psi$-related vector fields have $\Psi$-related flows together with $\Psi |_{\partial M}={\text {id}}$ readily implies that $(\Psi \circ \phi )(y,t)={\text {Fl}}^\xi _t(y)$. Using Sect. 3.4, and in particular Lemma 3.9, we see that we can complete the proof by showing that $\Psi \circ \phi \sim _{N+1}\phi$ as follows. By Lemma 3.9 it suffices to consider the pull backs of coordinate functions of local charts along these diffeomorphisms. We apply this to charts which are obtained by composing a product chart for $V\times [0,\epsilon )$ with $(\Psi \circ \phi )^{-1}$. Now the fact that $d\rho (\xi )\equiv 1$ (and that $\Psi \circ \phi$ maps $V\times \{0\}$ into $\partial M$) shows that applying this construction to the coordinate *t* on $[0,\epsilon )$, we obtain $\rho$. As observed above, $\rho \circ \Psi \sim _{N+1}\rho$ and thus $\rho \circ \Psi \circ \phi \sim _{N+1}\rho \circ \phi$. Thus it remains to consider functions *f* such that $f\circ (\Psi \circ \phi )$ is one of the boundary coordinate functions, and hence $\partial _t\cdot (f\circ \Psi \circ \phi )\equiv 0$ or, equivalently, $\xi \cdot f\equiv 0$ on an appropriate neighborhood of the boundary. Note then that $\phi ^*\xi \cdot (f\circ \phi )\equiv 0$. We have to compare $f\circ (\Psi \circ \phi )$ to $f\circ \phi$. As we have observed above, we get $\eta \sim _N\xi$ and hence $\phi ^*\xi \sim _N\phi ^*\eta =\partial _t$. Thus we get $\phi ^*\xi =\partial _t+t^N{{\tilde{\xi }}}$ for some vector field ${{\tilde{\xi }}}\in \mathfrak (V\times [0,\epsilon ))$. By construction $f\circ \phi$ and $f\circ (\Psi \circ \phi )$ agree on $V\times \{0\}$, so $f\circ \phi \sim _1f\circ (\Psi \circ \phi )$. Assuming that $f\circ \phi \sim _kf\circ (\Psi \circ \phi )$ for some $k\ge 1$, we get $f\circ \phi =f\circ (\Psi \circ \phi )+t^k{{\tilde{f}}}$ for some ${{\tilde{f}}}\in C^\infty (V\times [0,\epsilon ),{\mathbb {R}})$. Then we compute
  $$\begin{aligned} 0=(\partial _t+t^N{{\tilde{\xi }}})\cdot (f\circ (\Psi \circ \phi )+t^k\tilde{f})=0+kt^{k-1}{{\tilde{f}}}+{\mathcal {O}}(t^{\text {min}(k,N)}). \end{aligned}$$
  If $k\le N$, then this equation shows that ${{\tilde{f}}}$ vanishes along $V\times \{0\}$ and hence $(f\circ \phi )\sim _{k+1}f\circ (\Psi \circ \phi )$. Inductively, this gives $(f\circ \phi )\sim _{N+1}f\circ (\Psi \circ \phi )$, which completes the proof. $\square$

### Aligned metrics

Following an idea in [10] we next show that the freedom under diffeomorphisms asymptotic to the identity can be absorbed in a geometric relation between the metrics. The analogous condition in [10] is phrased as “transversality”.

#### Definition 3.12

Let ${\overline{M}}=M\cup \partial M$ be a manifold with boundary and let ${\mathcal {G}}$ be an equivalence class of ALH metrics for the relation $\sim _{N-2}$ for some $N\ge 3$. Consider two metrics $g,h\in {\mathcal {G}}$ and a local defining function $\rho$ for $\partial M$ defined on some open subset $U\subset {\overline{M}}$. Then we say that *h* *is aligned with* *g* *with respect to* $\rho$ if

$$\begin{aligned} \rho _ig^{ij}(h_{jk}-g_{jk})\equiv 0 \end{aligned}$$

on some open neighborhood of $U\cap \partial M$ in *U*.

Observe that the condition in Definition 3.12 can be rewritten as $\rho _ig^{ij}h_{jk}=\rho _k$. This in turn implies that $\rho _ih^{ij}=\rho _ig^{ij}$ and hence the gradients of $\rho$ with respect to the two metrics coincide on a neighborhood of the boundary. This shows that in the case that $\rho$ is adapted to $g_{ij}$ and $h_{ij}$ is aligned to $g_{ij}$ with respect to $\rho$, then $\rho$ is also adapted to $h_{ij}$.

#### Theorem 3.13

In our usual setting, of ${\overline{M}}=M\cup \partial M$ and a class ${\mathcal {G}}$ of metrics, assume that $g\in {\mathcal {G}}$ and $\rho$ is a local defining function for $\partial M$ that is adapted to *g*. Then for any $h\in {\mathcal {G}}$ and locally around any point $x\in \partial M$ in the domain of definition of $\rho$, there exists a diffeomorphism $\Psi$ such that $\Psi \sim _{N+1}{\text {id}}$ and such that $\Psi ^*h$ is aligned to *g* with respect to $\rho$. Moreover, the germ of $\Psi$ along the intersection of its domain of definition with $\partial M$ is uniquely determined by this condition.

#### Proof

Since $\rho$ is adapted to *g* the function $\rho ^{-2}g^{ij}\rho _i\rho _j$ is identically one on some neighborhood of the boundary. Now given $h_{ij}\in {\mathcal {G}}$, we can use Proposition 2.4 to modify $\rho$ to a local defining function *r* adapted to $h_{ij}$ in such a way that $\rho ^2g_{ij}$ and $r^2h_{ij}$ induce the same metric on the boundary, compare also to the proof of Lemma 3.10. Now we define vector fields $\xi =\xi ^i$ and $\eta =\eta ^i$ by $\xi ^i:=\rho ^{-2}g^{ij}\rho _j$ and $\eta ^i:=r^{-2}h^{ij}r_j$ where as usual we write $\rho _j$ for $d\rho$ and similarly for *r*. Recall from the proof of Theorem 3.11 that this implies that $\xi \sim _N\eta$.

Now, via the construction of collars from the proof of Theorem 3.11, we obtain a diffeomorphism $\Psi$ which has the property that for any *y* in an appropriate neighborhood of *x* in $\partial M$, we get $\Psi \circ {\text {Fl}}^\xi _t(y)={\text {Fl}}^\eta _t(y)$. Differentiating this equation shows that $\xi =\Psi ^*\eta$. By construction the derivative of the function $t\mapsto r({\text {Fl}}^\eta _t(y))$ is given by

$$\begin{aligned} dr(\eta )=r^{-2}r_ih^{ij}r_j\equiv 1, \end{aligned}$$

so $r(y)=0$ shows that $r({\text {Fl}}^\eta _t(y))=t$. In the same way $\rho ({\text {Fl}}^\xi _t(y))=t$, which shows that $\Psi ^*r=\rho$. Knowing this and $\xi \sim _N\eta$, the last part of the proof of Theorem 3.11 shows that $\Psi \sim _{N+1}{\text {id}}$.

To show that $\Psi$ has the required property, observe that by construction $r^2h_{ij}\eta ^j=r_i$. That is $i_\eta r^2h=dr$ and in the same way $i_\xi \rho ^2g=d\rho$. Using this and the above, we now obtain

$$\begin{aligned} i_\xi \rho ^2\Psi ^*h=i_{\Psi ^*\eta }\Psi ^*r^2h=\Psi ^*(i_\eta r^2h)=\Psi ^*(dr)=d\rho =i_\xi \rho ^2g. \end{aligned}$$

This shows that, if we insert $\xi$ into the bilinear form $\rho ^2(\Psi ^*h-g)$ (which by construction is smooth up to the boundary), the resulting one form vanishes identically on a neighborhood of the boundary. This exactly shows that $\Psi ^*h$ is aligned with *g* with respect to $\rho$, so the proof of existence is complete.

To prove uniqueness, assume that *h* is aligned to *g* with respect to $\rho$ and that a diffeomorphism $\Psi$ such that $\Psi \sim _{N+1}{\text {id}}$ has the property that also $\Psi ^*h$ is aligned to *g* with respect to $\rho$. Observe that $\Psi \sim _{N+1}{\text {id}}$ implies that $\Psi ^*h\in {\mathcal {G}}$. As observed above, the fact that both *h* and $\Psi ^*h$ are aligned with *g* with respect to $\rho$ implies that $\rho$ is adapted both to *h* and to $\Psi ^*h$. But on the other hand, the fact that $\rho$ is adapted to *h* of course implies that $\Psi ^*\rho$ is adapted to $\Psi ^*h$. Now by construction $\rho \circ \Psi \sim _{N+1}\rho$ and hence $(\rho \circ \Psi )\Psi ^*h$ and $\rho \Psi ^*h$ induce the same metric on the boundary. Hence the uniqueness part in Proposition 2.4 implies that $\rho \circ \Psi =\rho$ and hence $\Psi ^*\rho _i=\rho _i$ on a neighborhood of the boundary.

Now of course the inverse metric to $\Psi ^*h_{ij}$ is $\Psi ^*h^{ij}$ and $\Psi ^*\rho _i\Psi ^*h^{ij}=\rho _i\Psi ^*h^{ij}$. Since $\Psi ^*h_{ij}$ is aligned with $g_{ij}$ with respect to $\rho$, we get $\rho _i\Psi ^*h^{ij}=\rho _ig^{ij}$ and since also $h_{ij}$ is aligned with *g* with respect to $\rho$, $\rho _ig^{ij}$ equals $\rho _ih^{ij}$. Hence putting $\xi ^j:=\rho _ih^{ij}$ we conclude that $\Psi ^*\xi =\xi$. Since $\Psi$ is the identity on the boundary, this implies that $\Psi ({\text {Fl}}^\xi _t(y))={\text {Fl}}^\xi _t(y)$ for *y* in the boundary and *t* sufficiently small, so $\Psi ={\text {id}}$ locally around the boundary. $\square$

### The action of the aligning diffeomorphism

Fix $g\in {\mathcal {G}}$ and a local defining function $\rho$ for the boundary which is adapted to *g*. Consider another metric $h\in {\mathcal {G}}$ and the corresponding tensor field $\mu _{ij}$ defined by (2.1). From Theorem 3.13 we then know that locally around each boundary point, we find an essentially unique diffeomorphism $\Psi$ such that $\Psi \sim _{N+1}{\text {id}}$ and such that $\Psi ^*h$ is aligned with *g* with respect to $\rho$. Since $\Psi ^*h\in {\mathcal {G}}$, the analog of (2.1) defines a tensor field ${{\tilde{\mu }}}_{ij}$ that describes the difference between $\Psi ^*h$ and *g*. We now prove that the boundary value of ${{\tilde{\mu }}}_{ij}$ can be explicitly computed from the boundary value of $\mu _{ij}$. This will be the crucial step towards finding combinations of the two cocycles constructed in Sects. 3.1 and 3.2 which are invariant under diffeomorphisms asymptotic to the identity. Observe that this formally looks like the coordinate formula in Proposition 2.16 of [10], but the actual meaning is different: Our description does not involve any choice of local coordinates, but uses only abstract indices.

#### Theorem 3.14

In the setting of Theorem 3.13 for some fixed order *N*, let $\mu _{ij}$ be the tensor field relating $h_{ij}$ and $g_{ij}$ according to (2.1), and let ${{\tilde{\mu }}}_{ij}$ be the corresponding tensor field relating $\Psi ^*h_{ij}$ and $g_{ij}$. Then putting $\xi ^i:=\rho ^{-2}g^{ij}\rho _j$, we obtain

3.14 $$\begin{aligned} {{\tilde{\mu }}}_{ij}=\mu _{ij}-\rho _i\mu _{j\ell }\xi ^\ell -\rho _j\mu _{i\ell }\xi ^\ell + \tfrac{\xi ^k\mu _{k\ell }\xi ^\ell }{N}(\rho ^2g_{ij}+(N-1)\rho _i\rho _j)+\mathcal O(\rho ). \end{aligned}$$

#### Proof

We use the quantities introduced in the proof of Theorem 3.13: We denote by *r* the defining function adapted to $h_{ij}$ such that $r^2h_{ij}$ and $\rho ^2g_{ij}$ induce the same metric on the boundary. Further we put $\xi ^i:=\rho ^{-2}g^{ij}\rho _j$ and $\eta ^i:=r^{-2}h^{ij}r_j$. In terms of these, we know from the proof of Theorem 3.13 that the alignment diffeomorphism $\Psi$ satisfies $r\circ \Psi =\rho$ and $\Psi ^*\eta =\xi$, and hence $\Psi \circ {\text {Fl}}^\xi _t={\text {Fl}}^\eta _t$ wherever the flows are defined. Moreover, writing $r=e^v\rho$, we know from the proof of Lemma 3.10 that $v=\rho ^N{{\tilde{v}}}$ for a function ${{\tilde{v}}}$ that admits a smooth extension to the boundary. Moreover, we can compute the boundary value of ${{\tilde{v}}}$ from that proof: In equation (3.12) we can bring the first term on the right hand side to the left hand side and then use the fact that $\rho$ is adapted to *g* to rewrite (3.12) as

$$\begin{aligned} (\rho ^{-2}g^{ij}-\rho ^{-2}h^{ij})\rho _i\rho _j=2\rho \rho ^{-2}h^{ij}\rho _iv_j+\rho ^2\rho ^{-2}h^{ij}v_iv_j. \end{aligned}$$

In the left hand side, we can insert (2.4) and use the definition of $\xi$ to obtain $\rho ^N\xi ^k\mu _{k\ell }\xi ^\ell$. On the other hand $v_j=N\rho ^{N-1}{{\tilde{v}}}\rho _j+{\mathcal {O}}(\rho ^N)$. Inserting this in the right hand side and using that $\rho ^{-2}h^{ij}\rho _i\rho _j=1+{\mathcal {O}}(\rho )$, we obtain $2N\rho ^N{{\tilde{v}}}+{\mathcal {O}}(\rho ^{N+1})$, which shows that

3.15 $$\begin{aligned} {{\tilde{v}}}=\tfrac{1}{2N}\xi ^k\mu _{k\ell }\xi ^\ell +\mathcal O(\rho ). \end{aligned}$$

The basis for the further computation will be the fact that for vector fields $\zeta _1,\zeta _2$ (which we assume to be smooth up to the boundary), we get $\Psi ^*h(\Psi ^*\zeta _1,\Psi ^*\zeta _2)=h(\zeta _1,\zeta _2)\circ \Psi$. Multiplying by $\rho ^2$, we obtain

3.16 $$\begin{aligned} \rho ^2\Psi ^*h(\Psi ^*\zeta _1,\Psi ^*\zeta _2)=(r^2h(\zeta _1,\zeta _2))\circ \Psi , \end{aligned}$$

and both sides admit a smooth extension to the boundary. Hence the right hand side equals $r^2h(\zeta _1,\zeta _2)+{\mathcal {O}}(\rho ^{N+1})$. Inserting $r=\rho e^v$ and (2.1), we conclude that this equals

$$\begin{aligned} e^{2v}\rho ^2g(\zeta _1,\zeta _2)+e^{2v}\rho ^N\mu (\zeta _1,\zeta _2)+\mathcal O(\rho ^{N+1}). \end{aligned}$$

Of course, $e^{2v}=1+2\rho ^N{{\tilde{v}}}+{\mathcal {O}}(\rho ^{N+1})$ and we conclude that the right hand side of (3.16) equals

3.17 $$\begin{aligned} \rho ^2g(\zeta _1,\zeta _2)+\rho ^N\big (2{{\tilde{v}}}\rho ^2g(\zeta _1,\zeta _2)+ \mu (\zeta _1,\zeta _2)\big )+{\mathcal {O}}(\rho ^{N+1}). \end{aligned}$$

The left hand side of (3.16), by definition, can be written as $\rho ^2g(\Psi ^*\zeta _1,\Psi ^*\zeta _2)+\rho ^N{{\tilde{\mu }}}(\Psi ^*\zeta _1,\Psi ^*\zeta _2)$. Now we know that $\Psi ^*\zeta _1\sim _N\zeta _1$ and hence $\Psi ^*\zeta _1=\zeta _1+\rho ^N{{\tilde{\zeta }}}_1$, where ${{\tilde{\zeta }}}_1$ admits a smooth extension to the boundary, and similarly for $\zeta _2$. Inserting this, we obtain

3.18 $$\begin{aligned} \rho ^2g(\zeta _1,\zeta _2)+\rho ^N\big (\rho ^2g(\zeta _1,{{\tilde{\zeta }}}_2)+\rho ^2g({{\tilde{\zeta }}}_1,\zeta _2)+ {{\tilde{\mu }}}(\zeta _1,\zeta _2)\big )+{\mathcal {O}}(\rho ^{N+1}). \end{aligned}$$

Since this has to equal (3.17), we conclude that

3.19 $$\begin{aligned} {{\tilde{\mu }}}(\zeta _1,\zeta _2)=\mu (\zeta _1,\zeta _2)-\rho ^2g(\zeta _1,{{\tilde{\zeta }}}_2)- \rho ^2g({{\tilde{\zeta }}}_1,\zeta _2)+2{{\tilde{v}}}\rho ^2g(\zeta _1,\zeta _2)+\mathcal O(\rho ).\nonumber \\ \end{aligned}$$

The key observation now is that the computation of ${{\tilde{\zeta }}}_1$ and ${{\tilde{\zeta }}}_2$ essentially reduces to the computation of the vector field ${{\tilde{\eta }}}$ which has the property that $\eta =\xi +\rho ^N{{\tilde{\eta }}}$. As a first step, we claim that for a vector field $\zeta$ which is tangent to the boundary along the boundary, we have $\Psi ^*\zeta \sim _{N+1}\zeta$. This can of course be proved locally, so we can use local charts obtained from a collar construction as in the proof of Theorem 3.11. These have *r* as one coordinate and $\eta$ as the corresponding coordinate vector field. We first consider the case that $\zeta$ is the coordinate vector field $\partial _i$ associated to one of the boundary coordinates. Of course, $0=[\eta ,\partial _i]$ and pulling back along $\Psi$, we conclude that $0=[\xi ,\Psi ^*\partial _i]$. Now by Lemma 3.9, we know that $\xi \sim _N\eta$ and $\Psi ^*\partial _i\sim _N\partial _i$, and we express this via $\xi =\eta +r^N{{\tilde{\eta }}}$ and $\Psi ^*\partial _i=\partial _i+r^N{{\tilde{\zeta }}}$, where ${{\tilde{\eta }}}$ and ${{\tilde{\zeta }}}$ admit a smooth extension to the boundary. Plugging these expressions into the Lie bracket and using that $\partial _i\cdot r=0$ and $\eta \cdot r=1$, we conclude that

$$\begin{aligned} 0=[\xi ,\Psi ^*\partial _i]=[\eta ,\partial _i]+Nr^{N-1}{{\tilde{\zeta }}}+\mathcal O(r^N). \end{aligned}$$

This shows that ${{\tilde{\zeta }}}$ vanishes along the boundary and hence $\Psi ^*\partial _i\sim _{N+1}\partial _i$. Now a general vector field $\zeta$ that is tangent to the boundary along the boundary can be be written as $f\eta +\sum f_i\partial _i$ for arbitrary smooth functions $f_i$ and a smooth function *f* which is ${\mathcal {O}}(r)$. Thus the general version of our claim follows readily since $\Psi ^*\eta \sim _N\eta$, $f_i\circ \Psi \sim _{N+1}f_i$ and $f\circ \Psi \sim _{N+1}f$.

Now for any vector field $\zeta$ that is smooth up to the boundary, the difference $\zeta -d\rho (\zeta )\eta$ is smooth up to the boundary and tangent to the boundary along the boundary. Of course $\Psi ^*(d\rho (\zeta )\eta )=(d\rho (\zeta )\circ \Psi )\xi$ and so this equals $d\rho (\zeta )\xi +{\mathcal {O}}(\rho ^{N+1})$. Thus, writing $\zeta =d\rho (\zeta )\eta +(\zeta -d\rho (\zeta )\eta )$ and pulling back, we get

3.20 $$\begin{aligned} \rho ^N{{\tilde{\zeta }}}=\Psi ^*\zeta -\zeta =d\rho (\zeta )(\xi -\eta )+\mathcal O(\rho ^{N+1}). \end{aligned}$$

To compute the difference $\xi -\eta$, we first recall that $r_j=e^v\rho _j+e^v\rho v_j$ and $v_j=N\rho ^{N-1}{{\tilde{v}}}\rho _j+{\mathcal {O}}(\rho ^N)$. This shows that $r_j=\rho _j(1+(N+1)\rho ^N{{\tilde{v}}})+\mathcal O(\rho ^{N+1})$. Next, by definition $\eta ^i=e^{-2v}\rho ^{-2}h^{ij}r_j$ and

$$\begin{aligned} e^{-2v}(1+(N+1)\rho ^N{{\tilde{v}}})=1+(N-1)\rho ^N{{\tilde{v}}}+\mathcal O(\rho ^{N+1}). \end{aligned}$$

Now (2.4) shows that

$$\begin{aligned} \rho ^{-2}h^{ij}=\rho ^{-2}g^{ij}-\rho ^N(\rho ^{-2}g^{ik}\mu _{k\ell }\rho ^{-2}g^{\ell j})+{\mathcal {O}}(\rho ^{N+1}). \end{aligned}$$

Putting all this together, we see that

3.21 $$\begin{aligned} \eta ^j-\xi ^j=\rho ^N((N-1)\tilde{v}\xi ^j-\rho ^{-2}g^{jk}\mu _{k\ell }\xi ^\ell )+{\mathcal {O}}(\rho ^{N+1}). \end{aligned}$$

Dividing the negative of the right hand side by $\rho ^N$ and contracting with $\rho ^2g_{ij}$, we obtain $-(N-1)\tilde{v}\rho _j+\mu _{j\ell }\xi ^{\ell }$. Using this and (3.20), we can write (3.19) in abstract index notation as

3.22 $$\begin{aligned} {{\tilde{\mu }}}_{ij}=\mu _{ij}+2(N-1)\tilde{v}\rho _i\rho _j-\rho _i\mu _{j\ell }\xi ^\ell -\rho _j\mu _{i\ell }\xi ^\ell +2{{\tilde{v}}}\rho ^2g_{ij}+{\mathcal {O}}(\rho ), \end{aligned}$$

which together with (3.15) exactly gives the claimed formula. $\square$

Using this, we can easily deduce that appropriate combinations of the cocycles constructed in Sects. 3.1 and 3.2 remain unchanged if one of the two metrics involved is pulled back by a diffeomorphisms that is asymptotic to the identity. Since we are dealing with the situation of the classical mass here, we have to specialize to the case that $N=n$.

#### Corollary 3.15

In our usual setting of ${\overline{M}}=M\cup \partial M$, let ${\mathcal {G}}$ be an equivalence class of ALH metrics on *M* for the relation $\sim _{n-2}$. Let $c_1$ and $c_2$ be the cocycles constructed in Sects. 3.1 and 3.2, respectively, and let $c:=\tfrac{1}{n}c_1+\tfrac{1}{2}c_2$. Then *c* defines a cocycle on ${\mathcal {G}}$ that has the property that for metrics $g,h\in \mathcal G$ and any diffeomorphism $\Phi \in {\text {Diff}}_0^{n+1}({\overline{M}})$, we get $c(g,h)=c(g,\Phi ^*h)$.

#### Proof

We fix $g\in {\mathcal {G}}$ and a local defining function $\rho$ that is adapted to *g*. Then we show that for $c=\tfrac{1}{n}c_1+\tfrac{1}{2}c_2$ and the alignment diffeomorphism $\Psi$ obtained from Theorem 3.14, we get $c(g,\Psi ^*h)=c(g,h)$. The last part of Theorem 3.13 shows that $\Psi ^*h$ is the unique metric in the the orbit of *h* under ${\text {Diff}}_0^{n+1}({\overline{M}})$ which is aligned to *g* with respect to $\rho$. Hence applying the construction of Theorem 3.14 to $\Phi ^*h$ for arbitrary $\Phi \in {\text {Diff}}_0^{n+1}({\overline{M}})$, we also have to arrive at $\Psi ^*h$, which then implies the result.

Using the formulae in parts (2) of Propositions 3.1 and 3.5, we see that to prove our claim it suffices to show that the boundary value of

3.23 $$\begin{aligned} \tfrac{n^2-1}{2n}\rho ^{-2}g^{ij}\mu _{ij}-\tfrac{1}{2}\xi ^i\mu ^0_{ij}\xi ^j \end{aligned}$$

coincides with the boundary value of the analogous expression formed from ${{\tilde{\mu }}}_{ij}$. Inserting $\mu ^0_{ij}=\mu _{ij}-\tfrac{1}{n}\rho ^{-2}g^{k\ell }\mu _{k\ell }\rho ^2g_{ij}$ and using that $\xi ^i\rho ^2g_{ij}\xi ^j=1$ on a neighborhood of $\partial M$, we see that (3.23) equals

$$\begin{aligned} \tfrac{n}{2}\rho ^{-2}g^{ij}\mu _{ij}-\tfrac{1}{2}\xi ^i\mu _{ij}\xi ^j. \end{aligned}$$

Contracting $\rho ^{-2}g^{ij}$ into formula (3.14) (for the case $N=n$) and multiplying by $\tfrac{n}{2}$, we obtain

$$\begin{aligned} \tfrac{n}{2}\rho ^{-2}g^{ij}{{\tilde{\mu }}}_{ij}=\tfrac{n}{2}\rho ^{-2}g^{ij}\mu _{ij}- \tfrac{1}{2}\xi ^i\mu _{ij}\xi ^j. \end{aligned}$$

By alignment, $\xi ^i{{\tilde{\mu }}}_{ij}=0$, so this proves our claim. $\square$

#### Remark 3.16

The computations in this section can also be used to show that the cocycle from Corollary 3.15 simplifies in an important special case. Assume that we deal with two metrics $g_{ij}$ and $h_{ij}$ in $[{\mathcal {G}}]$ such that there is a local defining function $\rho$ which is adapted to both metrics at the same time. By definition, this implies $\rho _ih^{ij}\rho _j=\rho _ig^{ij}\rho _j$ on a neighborhood of the boundary. Assuming this, we can contract $\rho _i\rho _j$ into equation (2.4) and putting $\xi ^i=\rho ^{-2}g^{ij}\rho _j$ as above, the result reads as $0=\rho ^{N+2}\xi ^k\mu _{k\ell }\xi ^\ell +{\mathcal {O}}(\rho ^{N+3})$. Thus we conclude that $\xi ^k\mu _{k\ell }\xi ^\ell$ vanishes along the boundary. But in the proof of Corollary 3.15 we have seen that $\xi ^k\mu ^0_{k\ell }\xi ^\ell$ is a linear combination of $\xi ^k\mu _{k\ell }\xi ^{\ell }$ and of $\mu$. We have also seen there that our cocycle involves only the boundary values of $\xi ^k\mu ^0_{k\ell }\xi ^\ell$ and of $\mu$, so under the current assumptions this reduces to a multiple of $\mu$ only.

### From relative to absolute invariants

So far, we have not imposed any restriction on the equivalence class ${\mathcal {G}}$ of metrics beyond the fact that it consists of ALH-metrics. We next show that assuming that ${\mathcal {G}}$ locally contains metrics that are hyperbolic (i.e. have constant sectional curvature $-1$), one can use our construction to obtain an invariant for (single) metrics in ${\mathcal {G}}$. This assumption of course implies that the conformal infinity $[{\mathcal {G}}_\infty ]$ on $\partial M$ is conformally flat, but as we shall see below, it does not impose further restrictions on the topology of ${\overline{M}}$.

The key step toward this are results on the uniqueness of hyperbolic metrics with prescribed infinity that are discussed in Chapter 7 of [14]. These build on results in [21] and are related to the work in [13].

#### Theorem 3.17

Consider our usual setting, of ${\overline{M}}=M\cup \partial M$, and an equivalence class ${\mathcal {G}}$ of ALH metrics on *M* for the relation $\sim _{n-2}$. Assume that for each $x\in \partial M$ there is an open neighborhood *U* of *x* in ${\overline{M}}$ and a metric *g* in ${\mathcal {G}}$ that is hyperbolic (i.e. has constant sectional curvature $-1$) on *U*. Let *c* denote the cocycle from Corollary 3.15.

Then for an open subset *U* as above, and two metrics $g_1,g_2\in {\mathcal {G}}$ that are hyperbolic on *U*, we get $c(g_1,h)|_{U\cap \partial M}=c(g_2,h)|_{U\cap \partial M}$ for any $h\in {\mathcal {G}}$. Hence these quantities fit together to a well-defined section $c(h)\in \Omega ^{n-1}(\partial M,{\mathcal {T}}\partial M)$, thus defining a map *c* from ${\mathcal {G}}$ to tractor-valued differential forms. This is equivariant under diffeomorphisms preserving ${\mathcal {G}}$ in the sense that for $\Phi \in {\text {Diff}}_{{\mathcal {G}}}({\overline{M}})$, we obtain

$$\begin{aligned} c(\Phi ^*h)=(\Phi _\infty )^*c(h). \end{aligned}$$

Here $\Phi _{\infty }:=\Phi |_{\partial M}\in {\text {Conf}}(\partial M)$ and in the right hand side we use the standard action of conformal isometries on tractor-valued differential forms.

#### Proof

Suppose that $g_1,g_2\in {\mathcal {G}}$ are hyperbolic on *U*. Then we can apply Theorem 7.4 of [14] (see in particular the paragraph right after the proof of this theorem in [14]) to their restrictions to *U*. Observe also that the additional condition that is needed in Theorem 7.4 of [14], for the case $n=3$, is that the Schouten tensors of the two metrics in question agree along the boundary. For the rescalings of metrics in ${\mathcal {G}}$ which extend to the boundary, we have verified this in the end of Sect. 2.5. This implies that there is a neighborhood *V* of $U\cap \partial M$ in *U* and a diffeomorphism $\Psi :V\rightarrow V$ which restricts to the identity on $U\cap \partial M$ such that $g_1|_{V}=\Psi ^*(g_2|_V)$. Theorem 3.11 and Corollary 3.15 then immediately imply that $c(g_1,h)$ and $c(g_2,h)$ coincide on $U\cap \partial M$. It is then clear that we obtain the map *c* as claimed.

The equivariancy of *c* can be proved locally. So we take $\Phi \in {\text {Diff}}_{{\mathcal {G}}}({\overline{M}})$ and let $\Phi _\infty$ be its restriction to the boundary. Given $x\in \partial M$ we find an open neighborhood *U* of *x* in ${\overline{M}}$ and a metric $g\in {\mathcal {G}}$ such that $g|_U$ is hyperbolic. Now $\Phi ^{-1}(U)$ is an open neighborhood of $\Phi ^{-1}(x)$ in ${\overline{M}}$ and $\Phi ^*g|_{\Phi ^{-1}(U)}$ is hyperbolic on $\Phi ^{-1}(U)$. Thus we can compute $c(\Phi ^*h)$ as $c(\Phi ^*g,\Phi ^*h)$ on $\Phi ^{-1}(U)$, and by Proposition 3.7 this coincides with $(\Phi _\infty )^*(c(g,h)|_U)$. Since $c(g,h)|_U=c(h)|_U$, this implies the claim. $\square$

Suppose that $x\in \partial M$ and *U* is an open neighborhood of *x* in ${\overline{M}}$ such that ${\mathcal {G}}$ contains a metric *g* which is hyperbolic on *U*. Then of course the conformal class $[\mathcal G_\infty ]$ has to be flat on $U\cap \partial M$. In particular, the assumptions of Theorem 3.17 imply that $(\partial M,[{\mathcal {G}}_\infty ])$ is conformally flat, which in turn imposes restrictions on $\partial M$. However, if we are given a manifold ${\overline{M}}$ with boundary $\partial M$ and a flat conformal structure on $\partial M$, then there always is a class ${\mathcal {G}}$ of conformally compact metrics on *M*, for which the assumptions of Theorem 3.17 are satisfied, and hence we obtain an invariant for single metrics in ${\mathcal {G}}$.

Indeed, Proposition 7.2 of [14] (see also the discussion on p. 72 of that reference) shows that there is a hyperbolic metric *g* on some open neighborhood of $\partial M$ in ${\overline{M}}$ which induces the given boundary structure. Then of course *g* determines an equivalence class ${\mathcal {G}}$ of conformally compact ALH-metrics on *M* for which all the assumptions of Theorem 3.17 are satisfied.

We want to point out that it is not clear whether the condition of conformal flatness in Theorem 3.17 is of a fundamental nature. What one would need in more general situations is a class of “model metrics” in ${\mathcal {G}}$ which can be characterized well enough to obtain “uniqueness up to diffeomorphism” in a form as used in the proof of Theorem 3.17. An obvious idea is to assume that ${\mathcal {G}}$ contains at least one Einstein metric (which is a condition that is stable under diffeomorphism) and then look at appropriate classes of Einstein metrics in ${\mathcal {G}}$. In general, the Einstein condition is certainly not enough to pin down a metric up to diffeomorphism, compare with the non-uniqueness issues for the ambient metric [14]. However, it is well possible that there are situations in which additional (geometric) conditions can be imposed to ensure uniqueness.

### Recovering mass

We now show that in the special case of hyperbolic space, by an integration process of our cocycles, we can recover the mass for asymptotically hyperbolic metrics as introduced by Wang [22] and Chruściel-Herzlich [9]. In order to have an appropriate notion of integration available, we need the tractor bundle of the boundary to be globally trivialized by parallel sections. This essentially means that the boundary has to be a sphere. For other topologies, we can distill numerical global invariants out of our local invariant, but they cannot be expected to be as strong as a full “integral” of the local invariant to a tractor, see part (2) of Remark 3.19 below. So we specialize to the case that ${\overline{M}}$ is an open neighborhood of the boundary $S^{n-1}$ in the closed unit ball and that ${\mathcal {G}}$ is the equivalence class of (the restriction to *M* of) the Poincaré metric which we denote by *g* here. This of course implies that $[{\mathcal {G}}_\infty ]$ is the round conformal structure on $S^{n-1}$ and that ${\mathcal {G}}$ satisfies the conditions of Theorem 3.17. Hence we get a map $c:{\mathcal {G}}\rightarrow \Omega ^{n-1}(S^{n-1},\mathcal TS^{n-1})$ as described there.

If $n\ge 4$, conformal flatness of the round metric on $S^{n-1}$ implies that the tractor connection $\nabla ^{{\mathcal {T}}}$ is flat. Moreover, since $(\partial M,[{\mathcal {G}}_\infty ])$ is the homogeneous model of conformal structures, the tractor bundle ${\mathcal {T}}\partial M$ admits a global trivialization by parallel sections. This extends to the case $n=3$ with the tractor connection on $S^2$ constructed as discussed in Sect. 2.5. Indeed, since *g* is conformally flat and Einstein, the ambient tractor connection is flat and the scale tractor $I^A$ is parallel on all of ${\overline{M}}$. In view of Proposition 2.6, $I^A$ thus provides a parallel extension of the normal tractor $N^A$ off the boundary, and by [1] and e.g. Lemma 6.2 of [11], this implies that $\partial M$ is totally umbilic in ${\overline{M}}$. (Recall from Sect. 2.5 that the trace-free part of the second fundamental form is conformally invariant along $\partial M$, so being umbilic is a conformally invariant condition.) Hence the second fundamental form with respect to any metric conformal to *g* is pure trace. Using a scale with vanishing mean curvature, as in Sect. 2.5, the second fundamental form actually vanishes. Hence the ambient Levi Civita connection restricts to the Levi Civita connection on the boundary and by definition we use the restriction of the ambient Schouten tensor in the construction of the tractor connection on the boundary in Sect. 2.5. Hence formula (2.6) directly implies that the boundary tractor connection coincides with the restriction of the ambient tractor connection, so it is flat since the normal tractor is parallel. The trivialization by parallel sections then works exactly as in higher dimensions.

This easily implies that, fixing an orientation on $\partial M$, there is a well-defined integral that associates to each form $\omega \in \Omega ^{n-1}(\partial M, {\mathcal {T}} \partial M)$ a parallel section of ${\mathcal {T}}\partial M$. Indeed, on $\partial M$ we can take a global frame $\{s_i\}$ of ${\mathcal {T}} \partial M$ consisting of parallel sections, expand $\omega$ as $\sum _i\omega _is_i$ with $\omega _i\in \Omega ^{n-1}(\partial M)$ and then define $\int _{\partial M}\omega :=\sum _i(\int _{\partial M}\omega _i) s_i$. Of course, any other parallel frame consists of linear combinations of the $s_i$ with constant coefficients, so the result is independent of the choice of parallel frame.

Now the boundary tractor metric induces a tensorial map $\Omega ^{n-1}(\partial M,{\mathcal {T}} \partial M)\times \Gamma ({\mathcal {T}} \partial M ) \rightarrow \Omega ^{n-1}(\partial M)$, which we write as $(\omega ,s)\mapsto \langle \omega ,s\rangle$. Observe that the definition of the integral readily implies that for any parallel section $s\in \Gamma ({\mathcal {T}} \partial M)$ on $\partial M$, we obtain $\langle \int _{\partial M}\omega ,s\rangle =\int _{\partial M}\langle \omega ,s\rangle$. In particular, the coefficients of $\int _{\partial M}\omega$ with respect to a parallel frame can be computed as an ordinary integral over an $(n-1)$-form. Given a metric $h\in {\mathcal {G}}$, we can in particular apply this to *c*(*h*) and we will to show that, after appropriate normalization, $\int _{\partial M}c(h)$ recovers the mass of *h*.

We will work on ${\overline{M}}$ with the extension of the conformal class of *g*, which we again denote by [*g*]. Since *g* is conformally flat, this leads to a flat tractor connection and [*g*] restricts to $[{\mathcal {G}}_\infty ]$ on $\partial M$. Hence any parallel section $s\in \Gamma ({\mathcal {T}}\partial M)$ can be extended to a parallel section of ${\mathcal {T}}{\overline{M}}$ on a neighborhood of $\partial M$. (In fact, also ${\mathcal {T}}{\overline{M}}$ is globally trivialized by parallel sections, but we don’t really need this here.) Parallel sections of the standard tractor bundle are well understood and the approach to the standard tractor bundle in [1] is based on their relation to the conformal-to-Einstein operator from Remark 3.3. As discussed there, the conformal-to-Einstein operator is a conformally invariant differential operator which maps $\Gamma ({\mathcal {E}}[1])$ to $\Gamma ({\mathcal {E}}_{(ab)_0}[1])$. It is well known, see e.g. [1] that, in terms of the Levi-Civita connection and the Schouten tensor of any metric in the conformal class, this operator is given by

3.24 $$\begin{aligned} \tau \mapsto \nabla _{(a}\nabla _{b)_0}\tau +\textsf {P} _{(ab)_0}\tau \end{aligned}$$

It is also well known that if $\tau$ lies in the kernel of this operator, then on the complement of the zero locus of $\tau$, the metric $\tau ^{-2}{\textbf{g}}_{ab}$ determined by this scale is Einstein. Conversely, for any local Einstein metric in the conformal class the corresponding (local) scale lies in the kernel.

We have also discussed in Remark 3.3 that the operator (3.24) can be realized as a projection of $\nabla _a^{{\mathcal {T}}}D^A\tau$. So if $D^A\tau$ is a parallel for $\nabla ^{{\mathcal {T}}}$, the $\tau$ lies in the kernel of (3.24). Now it turns out (compare with [16]) that any parallel section of the tractor bundle is of the form $D^A\tau$, where $\tau$ is the projection of the tractor to $\Gamma ({\mathcal {E}}[1])$. Hence this projection and $D^A$ restrict to inverse bijections between parallel standard tractors and sections of ${\mathcal {E}}[1]$ that lie in the kernel of (3.24) (and the zero locus is automatically nowhere dense and plays no role in this interpretation).

Returning to our setting of the class [*g*] on ${\overline{M}}$, we denote by $\sigma \in \Gamma (\mathcal TM)$ the density corresponding to *g*. Since *g* is Einstein, $D^A\sigma$ is a parallel section of ${\mathcal {T}}{\overline{M}}|_M$ which thus extends to all of ${\overline{M}}$. From Propositions 2.5 and 2.6 the orthocomplement of its boundary value can be naturally identified with $\mathcal T\partial M$. On the other hand, on *M*, $\sigma$ is nowhere vanishing. Thus on *M*, we can write any parallel section of ${\mathcal {T}}{\overline{M}}$ as $D^A(V\sigma )$ for some smooth function $V:M\rightarrow {\mathbb {R}}$ such that $V\sigma$ lies in the kernel of (3.24). But in terms of the Levi-Civita connection of *g*, for which $\sigma$ is parallel, and taking into account that the Schouten tensor is pure trace, this is equivalent to vanishing of the trace-free part of $\nabla _a\nabla _b V$. To obtain parallel sections that lead to boundary tractors along $\partial M$, we can require in addition that $D^A(V\sigma )$ is orthogonal to $D^A\sigma$. Working in the scale determined by *g* and using formula (2.5), one immediately verifies that this condition is equivalent to requiring that *V*, in addition, satisfies $\Delta V=-2\textsf {P} V=nV$. The two required properties then can be equivalently encoded as a single equation, the KID (“Killing initial data”) equation (which naturally decomposes into trace-free part and trace-part)

3.25 $$\begin{aligned} \nabla _a\nabla _b V-g_{ab}\Delta V+(n-1)g_{ab}V=0. \end{aligned}$$

Hence we see that, via $\tfrac{1}{n} D^A(V\sigma )$ on *M*, solutions to this equations parameterize those parallel tractors on ${\overline{M}}$ which lie in ${\mathcal {T}}\partial M$ along $\partial M$, as recall ${\mathcal {T}}\partial M$  can be identified with ${N_A}^\perp \subset {\mathcal {T}} {\overline{M}}|_{\partial M}$. Put another way, solutions to (3.25) capture (in a 1–1 way) scales on *M* whose limit at $\partial M$ is an Einstein, or almost Einstein (in the sense of [11, 16]) metric on $\partial M$. But on the other hand, solutions to this equation parameterize the mass integrals in the classical approach to the AH version of mass, which was our original motivation for looking for a tractor description.

#### Theorem 3.18

Let ${\overline{M}}$ be an open neighborhood of $\partial M=S^{n-1}$ in the closed unit ball, let ${\mathcal {G}}$ be the equivalence class of the restriction of the hyperbolic (Poincaré) metric *g* to *M*. For a metric $h\in {\mathcal {G}}$ consider $c(h)\in \Omega ^{n-1}(\partial M,{\mathcal {T}}\partial M)$ as in Theorem 3.17. For a solution *V* of the KID equation (3.25), let $s_V\in \Gamma (\mathcal T\partial M)$ be the parallel section obtained as the boundary value of $\frac{1}{n} D^A(V\sigma )\in \Gamma ({\mathcal {T}}{\overline{M}})$ (with respect to [*g*]).

Then $-2\langle \int _{\partial M} c(h),s_V\rangle$ coincides with the mass integral associated to *V* in [9].

#### Proof

Using the conformal class [*g*] on ${\overline{M}}$ we have constructed $c(h)=c(g,h)$ as the boundary value of $\star _g\alpha$ for a certain ${\mathcal {T}}{\overline{M}}$-valued one-form $\alpha$ on *M*. Likewise, for a solution *V* of (3.25), the parallel boundary tractor $s_V$ is the restriction to $\partial M$ of a parallel section ${{\tilde{s}}}_V$ of ${\mathcal {T}}{\overline{M}}$. As we have noted already, $\langle \int _{\partial M} c(h),s_V\rangle =\int _{\partial M}\langle c(h),s_V\rangle$. This integrand is the boundary value of $\langle \star _g\alpha ,{\tilde{s}}_V\rangle$, which equals $\star _g\langle \alpha ,{{\tilde{s}}}_V\rangle$ by definition. Since this form is smooth up to the boundary, its integral over $\partial M$ equals the limit as $\epsilon \rightarrow 0$ of the integrals over the level sets $S_{\epsilon }=\{x:\rho (x)=\epsilon \}$. Since the mass integral associated to *V* is also expressed via a one-form, it suffices to compare that one-form to $\langle \alpha ,{{\tilde{s}}}_V\rangle$. In this comparison, we may work up to terms that vanish along the boundary after application of $\star _g$ and hence up to ${\mathcal {O}}(\rho ^{n-1})$, cf. the proof of Proposition 3.1.

We use the description of the mass integral associated to *V* from [19], it is shown in that reference that this agrees with the original mass integral introduces in [9]. The mass integrand associated to a solution *V* of the KID equation (3.25) is given by

$$\begin{aligned} V(\nabla ^i\lambda _{ia}-\nabla _a{\text {tr}}(\lambda ))-g^{ij}\lambda _{ia}\nabla _jV+ {\text {tr}}(\lambda )\nabla _aV, \end{aligned}$$

where $\lambda _{ij}=h_{ij}-g_{ij}$, the hyperbolic metric *g* is used to raise and lower indices and to form traces, and $\nabla$ is the Levi-Civita connection of *g*. Decomposing $\lambda _{ij}=\lambda ^0_{ij}+\frac{1}{n}{\text {tr}}(\lambda )g_{ij}$, this becomes

3.26 $$\begin{aligned} (V\nabla ^i\lambda ^0_{ia}-\lambda ^0_{ia}\nabla ^iV)+ \tfrac{n-1}{n}({\text {tr}}(\lambda )\nabla _aV-V\nabla _a{\text {tr}}(\lambda )). \end{aligned}$$

Choosing a defining function $\rho$ adapted to $g_{ij}$, we then obtain $\lambda ^0_{ij}=\rho ^{n-2}\mu ^0_{ij}$ and ${\text {tr}}(\lambda )=\rho ^n\mu$ for the quantities introduced in and below equation (2.1) (for $N=n$). Note that $\mu ^0_{ij}$ and $\mu$ are smooth up to the boundary. We also know from above that $\sigma V$ is the projection of the parallel tractor ${{\tilde{s}}}_V=\frac{1}{n}D(\sigma V)$, so this is smooth up to the boundary. Since $\sigma$ is a defining density for $\partial M$, it follows that $\rho V$ is smooth up to the boundary.

Now we analyze the two parts of (3.26) separately, starting with the part involving ${\text {tr}}(\lambda )$. Here we have the advantage that covariant derivatives are only applied to smooth functions (and not to tensor fields), so the fact that $\nabla$ is not smooth up to the boundary does not matter. Since $\rho V$ is smooth up to the boundary, $\rho \nabla _a\rho V$ is ${\mathcal {O}}(\rho )$. Writing $\rho _a$ for $d\rho$ as before, we compute this as $\rho _a\rho V+\rho ^2\nabla _aV$. Thus $\rho ^2\nabla _aV=-\rho _a\rho V+\mathcal O(\rho )$ and in particular is smooth up to the boundary. Using this, we obtain

$$\begin{aligned} {\text {tr}}(\lambda )\nabla _aV=\rho ^n\mu \nabla _aV=-\rho ^{n-2}\rho _a\rho V\mu +{\mathcal {O}}(\rho ^{n-1}). \end{aligned}$$

Similarly, $V\nabla _a{\text {tr}}(\lambda )=V\nabla _a\rho ^n\mu =n\rho ^{n-2}(\rho V)\rho _a\mu +O(\rho ^{n-1})$. Hence the second part in (3.26) simply gives $-\frac{n^2-1}{n}\rho ^{n-2}(V\rho )\rho _a\mu +O(\rho ^{n-1})$.

Analyzing the second summand in the first part of (3.26) is similarly easy. This writes as

$$\begin{aligned} -\rho ^{-2} g^{ij}\rho ^n\mu ^0_{ia}\nabla _jV=\rho ^{n-2} \rho ^{-2} g^{ij}\rho _j\mu ^0_{ia}(\rho V)+{\mathcal {O}}(\rho ^{n-1}). \end{aligned}$$

For the first summand in (3.26), the analysis is slightly more complicated. This can be written $V\rho \rho ^{-2}g^{ij}\rho \nabla _i\rho ^{n-2}\mu ^0_{ja}$ and hence equals

3.27 $$\begin{aligned} V\rho \rho ^{-2}g^{ij}\rho ^{n-2}((n-2)\rho _i\mu ^0_{ja}+\rho \nabla _i\mu ^0_{ja}). \end{aligned}$$

Since in the last summand, we apply a covariant derivative to a tensor field, we have to change to a connection that admits a smooth extension to the boundary in order to analyze the boundary behavior. Hence we change from the Levi-Civita connection $\nabla$ of $g_{ij}$ to the Levi-Civita connection ${{\bar{\nabla }}}$ of $\bar{g}_{ij}:=\rho ^2g_{ij}$, which has this property. For the usual conventions, as used in [1], the one form $\Upsilon _a$ associated to this conformal change is given by $\Upsilon _a=\tfrac{\rho _a}{\rho }$. The relevant formula for the change of connection is then given by

$$\begin{aligned} \nabla _i\mu ^0_{ja}={{\bar{\nabla }}}_i\mu ^0_{ja}+2\Upsilon _i\mu ^0_{ja}+\Upsilon _j\mu ^0_{ia}+ \Upsilon _a\mu ^0_{ij}-\Upsilon _k{{\bar{g}}}^{k\ell }\mu ^0_{\ell a}\bar{g}_{ij}-\Upsilon _k{{\bar{g}}}^{k\ell }\mu ^0_{j\ell }{{\bar{g}}}_{ia}, \end{aligned}$$

This immediately shows that $\rho \nabla _i\mu ^0_{ja}$ admits a smooth extension to the boundary and its boundary value can be obtained by dropping the first summand in the right hand side of this formula and replacing each occurrence of $\Upsilon$ in the remaining terms by $d\rho$, so $\Upsilon _i$ becomes $\rho _i$ and so on. Inserting this back into (3.27), we get a contraction with ${{\bar{g}}}^{ij}$. This kills the term involving $\mu ^0_{ij}$ by trace-freeness, while all other terms become multiples of ${{\bar{g}}}^{ij}\rho _i\mu ^0_{ja}$. The factors of the individual terms are 2, 1, $-n$, and $-1$, respectively, so we’ll get a total contribution of $(2-n){\bar{g}}^{ij}\rho _i\mu ^0_{ja}$. This actually implies that (3.27) is ${\mathcal {O}}(\rho ^{n-1})$. Hence we finally conclude that (3.26) equals

3.28 $$\begin{aligned} \rho ^{n-2}(\rho V)\left( \rho ^{-2}g^{ij}\rho _j\mu ^0_{ia}- \tfrac{n^2-1}{n}\rho _a\mu \right) +O(\rho ^{n-1}). \end{aligned}$$

Now recall the formula for $\nabla ^{{\mathcal {T}}}_bD^A(\tau -\sigma )$ from part (1) of Proposition 3.1, taking into account the definition of ${\textbf{X}}^A$. This shows that, up to $\mathcal O(\rho ^{n-1})$, $\nabla ^{{\mathcal {T}}}_bD^A(\tau -\sigma )$ is given by inserting $\tfrac{n^2-1}{2}\rho ^{n-2}\rho _b\mu \rho \sigma ^{-1}$ into the bottom slot of a tractor. Pairing this with $\frac{1}{n}D^B(\sigma V)$ using the tractor metric, we simply simply obtain the product of $\sigma V$ with this bottom slot, i.e.

$$\begin{aligned} \tfrac{n^2-1}{2}\rho ^{n-2}\rho _b\mu (\rho V)+{\mathcal {O}}(\rho ^{n-1}). \end{aligned}$$

Analyzing the formula for $S(\sigma (h_{ij}-g_{ij})^0)$ from part (1) of Proposition 3.5 we similarly see that the pairing of this with $\frac{1}{n}D^B(\sigma V)$ gives

$$\begin{aligned} -\rho ^{n-2}\rho ^{-2}g^{ij}\rho _j\mu ^0_{ia}(\rho V)+\mathcal O(\rho ^{n-1}). \end{aligned}$$

But this exactly tells us that, up to ${\mathcal {O}}(\rho ^{n-1})$, (3.28) equals $-2\langle \alpha ,{{\tilde{s}}}_V\rangle$, where $\alpha$ corresponds to $c= \frac{1}{n}c_1+\frac{1}{2}c_2$, as in Corollary 3.15. $\square$

#### Remark 3.19

1. The mass integrals in [9], that we compare our cocycle to, are known to reproduce the definition of mass by Wang in [22], assuming the stronger conditions on asymptotics used there. In [22] the components of a mass vector are obtained from integrals that involve (in our language) only the trace of *h* with respect to *g*. The reason why, in our approach, also the trace-free part of the difference of the two metrics is needed is explained by Remark 3.16. Indeed the basic setup of Wang assumes (by an appropriate choice of coordinates) that there is a boundary defining function which is adapted both to *h* and to the background metric *g*.
2. As mentioned at the beginning of Sect. 3.9, we need the global trivialization of the boundary tractor bundle in order to define an integral of the invariant *c*(*h*) as a parallel section of ${\mathcal {T}}\partial M$. So this does not work for example if the boundary $\partial M$ is a torus, where most of the local parallel sections of ${\mathcal {T}}\partial M$ do not extend to global parallel sections. We can form an integral quantity associated to a global parallel section of ${\mathcal {T}}\partial M$ which is the boundary value of the tractor $s_V$ associated to a solution *V* of the KID equation as above by forming $-2\int _{\partial M}\langle c(h),s\rangle$. These integral quantities are sometimes referred to as mass in such situations. However, we want to emphasize that they contain only partial information about *c*(*h*), since they see only the projection of *c*(*h*) to the subbundle spanned by parallel tractors. We believe that in such situations one should try to work with the full invariant *c*(*h*) rather than just with the integrals against parallel sections. In some cases very useful information is likely contained in these “partial” mass quantities, but understanding this fully we leave as a direction for future research.

## Data Availability

Data availability is not applicable to this article as no new data were created or analyzed in this study.
